# Hypoconnectivity of Resting-State Networks in Persons with Aphasia Compared with Healthy Age-Matched Adults

**DOI:** 10.3389/fnhum.2017.00091

**Published:** 2017-02-28

**Authors:** Chaleece W. Sandberg

**Affiliations:** Adult Neuroplasticity Laboratory, Department of Communication Sciences and Disorders, Penn State UniversityUniversity Park, PA, USA

**Keywords:** aphasia, fMRI, resting state, functional connectivity, resting state networks, stroke recovery, language recovery

## Abstract

Aphasia is a language disorder affecting more than one million people in the US. While language function has traditionally been the focus of neuroimaging research, other cognitive functions are affected in this population, which has implications not only for those specific processes but also for the interaction of language and other cognitive functions. Resting state fMRI (rs-fMRI) is a practical and informative way to explore and characterize general cognitive engagement and/or health in this population, but it is currently underutilized. The aim of this study was to explore the functional connectivity in resting state networks (RSNs) and in the semantic network in seven persons with aphasia (PWA) who were at least 6 months post onset compared with 11 neurologically healthy adults (NHA) in order to gain a more comprehensive understanding of general cognitive engagement in aphasia. These preliminary results show that PWA exhibit hypoconnectivity in the semantic network and all RSNs except the visual network. Compared with NHA, PWA appear to have fewer cross- and left-hemispheric connections. However, PWA exhibit some stronger connections than NHA within the semantic network, which could indicate compensatory mechanisms. Importantly, connectivity for RSNs appear to increase with decreasing aphasia severity and decrease with increasing lesion size. This knowledge has the potential to improve aphasia therapy by furthering the understanding of lesion effects on the cognitive system as a whole, which can guide treatment target selection and promotion of favorable neural reorganization for optimal recovery of function.

## Introduction

The intrinsic activity of the brain has emerged as an informative way to characterize general cognitive engagement in healthy and clinical populations. The brain's intrinsic activity is typically captured during a resting state fMRI (rs-fMRI) scan, in which the participant is not performing any specific task for several minutes. In healthy brains, several resting state networks (RSNs) have been identified.

RSNs are sets of brain regions whose BOLD activation patterns have been shown to be reliably synchronous over time while the brain is not actively engaged in a specific task. These networks, as outlined by Raichle in recent reviews (Raichle, 2011, 2015), include the Default Mode Network (DMN), the Executive Control Network (ECN), the Auditory Network, the Visual Network, the Sensorimotor Network (SMN), the Salience Network, and the Dorsal Attention Network (DAN). The finding that spontaneous neural activity is organized by cognitive function allows for the comparison of neural organization between normal and disordered populations, unencumbered by the nuances of task design and task demands.

The brain's intrinsic activity has been shown to be disrupted in predictable ways in neurogenic disorders such as Alzheimer's disease, making rs-fMRI a plausible candidate as a biomarker for certain disorders (Fox and Greicius, 2010). However, less is known regarding the effect of lesioned tissue on intrinsic activity. Studies examining rs-fMRI in traumatic brain injury (TBI) and stroke have begun to emerge. For example, Hillary et al. (2014) found patterns of hyperconnectivity in the salience network, ECN, and DMN during rest in persons with TBI. Carter et al. (2010) found that, within 4 weeks after stroke, decreased interhemispheric connectivity of homologs within the DAN correlated with worse performance on a spatial attention task. Tuladhar et al. (2013) found reduced DMN connectivity in stroke patients compared with healthy controls.

Further, some studies have examined the specific effect of focal lesions on network organization during rest. For example, using a sample of 35 stroke, TBI, and tumor patients, Gratton et al. (2012) examined the effect of focal damage on network modularity (how regions cluster together into subnetworks) during rest. They found that focal damage to regions which connect modules (subnetworks) disrupts the modular organization of the brain, even in the undamaged hemisphere. On the other hand, damage to network hubs (regions central to network modules) did not disrupt the modular organization of the brain. Similarly, Eldaief et al. (2016) examined the effect of focal damage to the medial prefrontal cortex, which is a hub of the DMN, but also has links with other networks. They found that functional connectivity within the DMN was not affected, but that functional couplings between the DMN and other RSNs were affected. The results of these studies point to the effect focal lesions may have on global functioning.

Taken together, these studies suggest that rs-fMRI is a practical and informative way to explore and characterize general cognitive engagement and neural health in patient populations with neurogenic disorders. Further, the ease with which rs-fMRI is acquired makes it an attractive methodology for use in a population such as persons with aphasia (PWA), who, depending on severity, may be limited in the tasks they are able to perform in an MRI machine. However, rs-fMRI is currently underutilized in aphasia research.

Aphasia is a language disorder resulting from acquired brain injury, such as stroke, that affects more than one million people in the US (National Aphasia Association). While aphasia has traditionally been considered a language-based deficit, there is a growing awareness among clinicians and clinical aphasiologists that PWA experience deficits in other cognitive functions, including attention (e.g., Murray, 2000; Heuer and Hallowell, 2015; Villard and Kiran, 2015), executive function (e.g., Purdy, 2002; Fridriksson et al., 2006), and nonverbal working memory (e.g., Christensen and Wright, 2010; Potagas et al., 2011; Mayer and Murray, 2012). However, there is a paucity of neuroimaging research exploring these cognitive functions in aphasia (e.g., Crosson et al., 2005; Brownsett et al., 2014; Butler et al., 2014). It is possible that these deficits are related to disorganization or hypoconnectivity of the networks that support these extralinguistic cognitive functions. One way to explore this possibility is by examining the integrity of the RSNs using rs-fMRI in aphasia.

However, only a handful of studies to date have explored rs-fMRI in aphasia. Importantly, the majority of these studies focused on early stages after stroke and each study explored a different network or set of regions. For example, Yang et al. (2016) found increased amplitude of low-frequency fluctuations (ALFF) during rest in right temporal regions and decreased ALFF in left frontal regions in PWA at ~9 days post onset compared with a healthy control group. Zhu et al. (2014) found hypoconnectivity in the left frontoparietal network of PWA at 1 month post onset compared to a healthy control group. Wang et al. (2014) found both increased and decreased functional connectivity in the DMN in PWA compared with a healthy control group. Nair et al. (2015) found decreased connectivity in the language network in left hemisphere stroke patients (without aphasia, but with worse language performance than controls) at 5 days post onset compared with a healthy control group. Two of these studies were longitudinal in nature and found increases in connectivity at 2 months (Zhu et al., 2014) and 4.5 months (Nair et al., 2015) that coincided with behavioral improvement. Relatedly, van Hees et al. (2014) explored both ALFF and functional connectivity of rs-fMRI in eight chronic PWA before and after targeted therapy for word finding compared with a healthy control group. They found differences between groups pretreatment and changes in rs-fMRI in selected language regions that coincided with behavioral improvement in PWA.

Importantly, no study to date has systematically examined functional connectivity within each RSN in chronic aphasia compared with healthy controls. Thus, the purpose of this study was to analyze the functional connectivity within each RSN described by Raichle (2011) in PWA who were in the chronic stage of recovery compared with neurologically healthy adults (NHA). Additionally, because the population of interest is one for which word-finding difficulty is a ubiquitous feature, the semantic network, based on a meta-analysis and review of semantic processing (Binder et al., 2009; Price, 2012), was also explored.

The hypothesis for this study was that PWA would exhibit altered connectivity within both the semantic network and traditional RSNs when compared with NHA, and that this altered connectivity would largely reflect reduced connectivity. This hypothesis is based on the available evidence of brain connectivity in PWA at rest, which suggests differences between PWA and NHA, with an overall pattern of reduced connectivity, especially in left hemisphere regions. Exceptions to this pattern exist—i.e., increased connectivity for PWA—and may indicate compensatory mechanisms. Networks that include regions which are routinely lesioned in PWA should be especially susceptible to reduced connectivity. Correspondingly, aphasia severity and lesion size should also coincide with reduced connectivity, especially in the left hemisphere.

## Methods

### Participants

Seven (five male, two female) PWA and eleven (six male, five female) NHA were recruited. All participants were right handed and had completed at least a high school education. An MRI safety screening was conducted to ensure that each participant was safe to enter the bore of the magnet.

#### Persons with aphasia

**Figure 1:**
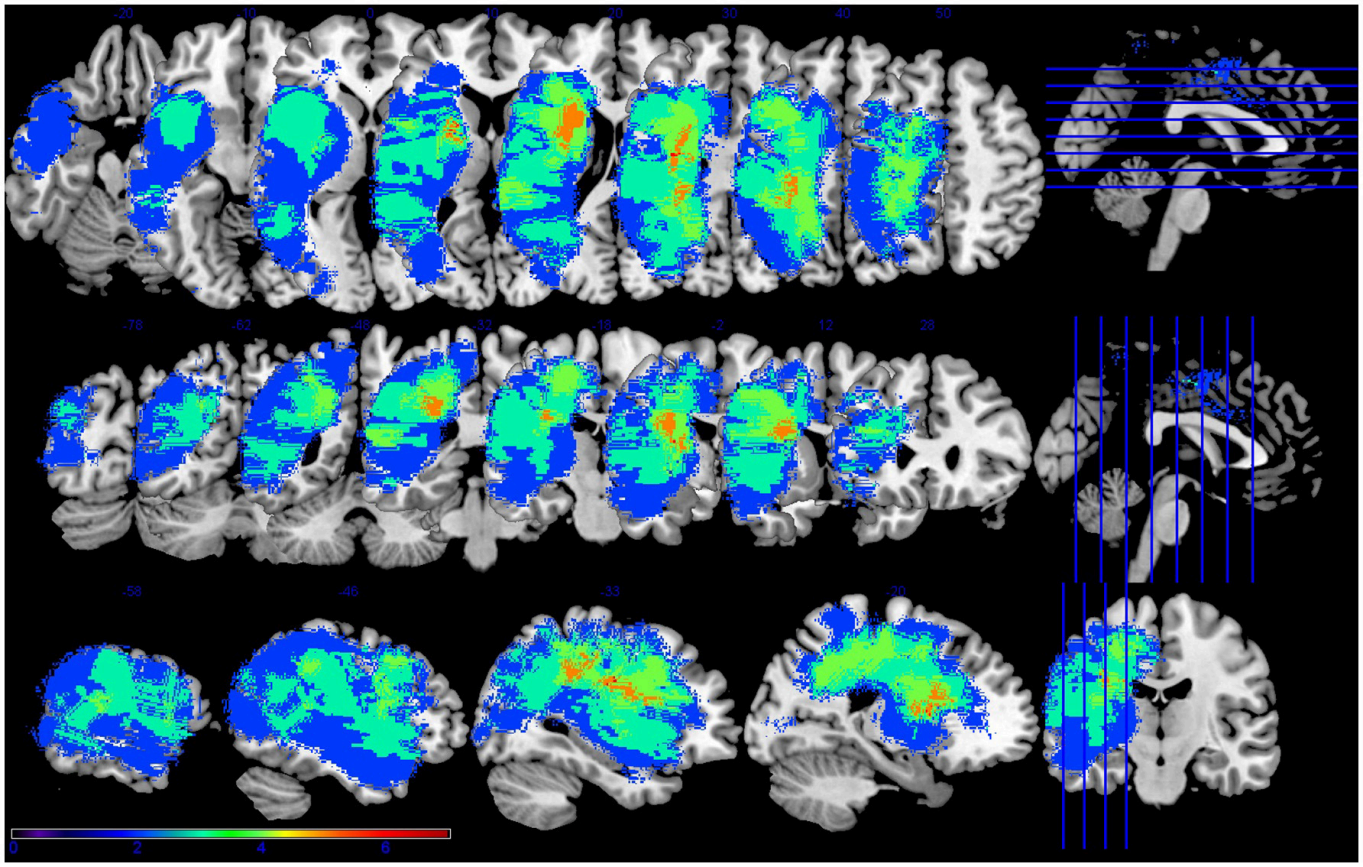
**Lesion overlap for the seven participants with aphasia**. Figure presented following neurological convention with left visualized on the left. Warm colors represent greater overlap and cool colors represent less overlap. The regions with the most overlap were in the internal capsule and corona radiata of the left hemisphere.

PWA were aged 47–75 (*M* = 60, *SD* = 12), had sustained a cerebrovascular accident in the left middle cerebral artery, were in the chronic stage of post-stroke recovery (at least 6 months post onset), and had no developmental or additional acquired neurogenic disorders. PWA completed a battery of standardized language tests to establish type and severity of aphasia as well as to provide a complete linguistic profile. These tests included the Western Aphasia Battery (WAB-R; Kertesz, 2006), the Boston Naming Test (BNT; Goodglass et al., 1983), selected subtests (written semantic association, auditory and written lexical decision, auditory and written synonym judgment) of the Psycholinguistic Assessment of Language Processing in Aphasia (PALPA; Kay et al., 1992), and selected subtests (three words, three pictures) of the Pyramids and Palm Trees (PAPT; Howard and Patterson, 1992). PWA also completed the Cognitive Linguistic Quick Test (CLQT; Helm-Estabrooks, 2001) to determine the relative contribution of cognitive deficits such as attention and memory to language dysfunction. See Table 1 for demographic information and Figure 1 for lesion overlap data. PWA gave informed consent according to the procedures approved by Boston University Institutional Review Board.

**Table 1:** **Demographic information for all participants**.

**Neurologically healthy adults**	**NHA01**	**NHA02**	**NHA03**	**NHA04**	**NHA05**	**NHA06**	**NHA07**	**NHA08**	**NHA09**	**NHA10**	**NHA11**
Gender	Female	Male	Male	Female	Female	Male	Male	Male	Female	Female	Male
Age	64	66	47	55	72	74	72	57	59	61	56
CLQT	WNL	WNL	WNL	WNL	WNL	WNL	WNL	WNL	WNL	WNL	WNL
**Persons with aphasia**	**PWA01**	**PWA02**	**PWA03**	**PWA04**	**PWA05**	**PWA06**	**PWA07**				
Gender	Male	Male	Male	Female	Male	Male	Female				
Age	47	53	48	74	69	75	56				
Months Post Onset	42	117	93	134	16	11	7				
Lesion Size (cc)	14.24	163.12	255.95	101.06	0.33	3.54	108.64				
Territory	Left MCA	Left MCA	Left MCA	Left MCA	Left MCA	Left MCA	Left MCA				
Aphasia Type	Anomic	Broca's	Conduction	Anomic	Anomic	TCM	Anomic				
Aphasia Severity (WAB AQ)	95.5	41.7	72.5	90.8	97.1	84.7	67.4				
Boston Naming Test	95%	22%^*^	82%^*^	68%^*^	95%	90%	90%				
**PALPA**											
Lexical Decision	97%	96%	98%	98%	99%	99%	99%				
Synonym Judgment	97%	77%	89%	93%	97%	100%	97%				
Semantic Association	77%	33%^*^	73%	80%	90%	90%	83%				
**PYRAMIDS AND PALM TREES**											
Pictures	100%	88%^*^	90%	77%^*^	98%	98%	96%				
Written Words	98%	85%^*^	96%	94%	100%	96%	69%^*^				
CLQT	WNL	mild	mild	mild	mild	WNL	mild				

WNL, within normal limits; CLQT, Cognitive Linguistic Quick Test; MCA, middle cerebral artery; TCM, transcortical motor; WAB AQ, Western Aphasia Battery Aphasia Quotient; PALPA, Psycholinguistic Assessment of Language Processing in Aphasia. Because scores were similar between auditory and written versions of the PALPA subtests, the presented score is an average of the two modalities. For the BNT, ^*^ = below 10th percentile of Tombaugh and Hubley (1997) norms. For PALPA, ^*^ = more than 2SD below mean of available norms (Synonym Judgment not available). For PAPT, ^*^ = below 90% cutoff for WNL performance.

#### Neurologically healthy adults

NHA were aged 47–74 (*M* = 62, *SD* = 8) and had no history of developmental or acquired neurogenic disorders. See Table 1 for demographic information. A cognitive screening using the CLQT was conducted to confirm absence of cognitive impairment. NHA gave informed consent according to the procedures approved by Penn State University Institutional Review Board, MRI data acquisition.

All participants completed a 6 min resting state scan (Van Dijk et al., 2010), with instructions to remain still, stay awake and look at the stimulus. The visual stimulus was a white dot on a black background presented on a screen behind the scanner, which projected to a mirror fitted to the head coil. Padding was used to minimize head motion and corrective optical lenses were used when necessary to correct visual acuity. After bore entry, the magnet was shimmed to achieve maximum homogeneity.

#### PWA

PWA were scanned at the Boston University Center for Biomedical Imaging in a 3 Tesla Philips Achieva MRI scanner with an 8-channel head coil that has been approved for use on humans. High-resolution T1-weighted images were acquired with the following parameters: 140 sagittal slices, 1 mm^3^ voxels, base resolution = 240 × 240, flip angle = 8°, phase encoding = AP, TR = 8.2 ms, TE = 3.8 ms. Blood-oxygen-level-dependent (BOLD) sensitive functional images were collected using the following parameters: 48 axial slices (3.3 mm thick), 3.3 mm^3^ voxels, base resolution = 64 × 59, flip angle = 80°, phase encoding = AP, TR = 3000 ms, TE = 30 ms.

#### NHA

NHA were scanned at the Penn State University Social, Life, and Engineering Sciences Imaging Center (SLEIC) in a 3 Tesla Siemens Magnetom Prisma Fit MRI scanner with a 20-channel head coil that has been approved for use on humans. High-resolution T1-weighted images were acquired with the following parameters: 160 sagittal slices, 1 mm^3^ voxels, base resolution = 256 × 256, flip angle = 9°, phase encoding = AP, TR = 1650 ms, TE = 2.03 ms. Blood-oxygen-level-dependent (BOLD) sensitive functional images were collected using the following parameters: 42 axial slices (3 mm thick), 3 mm^3^ voxels, base resolution 80 × 80, flip angle = 90°, phase encoding = AP, TR = 2500 ms, TE = 25 ms.

### Data analysis

The resting-state fMRI (rs-fMRI) data were analyzed using the CONN toolbox for SPM12 (Whitfield-Gabrieli and Nieto-Castanon, 2012).

#### Preprocessing

The rs-fMRI data were preprocessed using a standard SPM12 pipeline: functional volume realignment and unwarping, slice timing correction, segmentation, and normalization of structural volumes to the MNI template, normalization of functional volumes, and smoothing of functional volumes with a 5 mm FWHM kernel. The CONN toolbox additionally implements ART (ARTifact detection tools, www.nitrc.org/projects/artifact_detect) during the preprocessing pipeline to detect motion outliers, as head motion is especially confounding for resting state data (Van Dijk et al., 2012). The ART parameters consisted of a global signal threshold of three standard deviations away from the mean, a linear motion threshold of 0.5 mm, and a rotation threshold of 0.02 radians. After the segmentation step, PWA scans were visually checked by the author to verify lesion segmentation into the CSF mask. This classifies the lesion as a noise source so it does not contribute noisy data to the effects of interest. PWA scans were also visually checked after the normalization step to rule out any skewing effects of the lesion. After preprocessing, the CONN toolbox implements a denoising step using the CompCor method (Behzadi et al., 2007). A band-pass filter of 0.008–0.09 Hz was used to pass the low-frequency fluctuations of interest, linear trends were removed, and a linear regression of noise sources—e.g., white matter and CSF, six rigid body head motion parameters, motion outliers, and the main effect of the condition (rest)—was performed.

Lesion maps were hand-drawn by the author on the structural image in native space using MRIcron (http://people.cas.sc.edu/rorden/mricron/) and binarized. These maps were used to determine lesion overlap for the group, percent tissue spared for each anatomical region of interest (ROI) within each RSN for each PWA, and for lesion volume calculation for inclusion as a covariate of interest in the functional connectivity analyses.

#### Statistical analysis

Functional connectivity within eight resting-state networks (RSNs) was analyzed for each group. The DMN was defined using the atlas provided by CONN based on work by Fox et al. (2005), which used 12 mm spheres centered on published foci. The dorsal attention, executive control, salience, sensorimotor, visual, and auditory networks were defined based on Raichle (2011). The semantic network was defined based on a meta-analysis of semantic processing imaging studies and a review of imaging studies of language processing (Binder et al., 2009; Price, 2012). The anatomical ROIs for each RSN were defined using the regional boundaries within which the published foci lie in the default map in the CONN toolbox, which utilizes the Harvard-Oxford atlas for cortical and subcortical regions. See Table 2 for a list of regions, their abbreviations, and MNI coordinates within each network.

**Table 2:** **Regions of interest names and MNI coordinates used for each resting state network analysis**.

**Network**	**Region (as named in each atlas)**	**Abbreviations**	**MNI *x, y, z* coordinates**
DMN	Medial prefrontal cortex	MPFC	−1, 49, −5
	Posterior cingulate cortex	PCC	−6, −52, 40
	Left/right lateral parietal	LLP/RLP	−46, −70, 36/46, −70, 36
Semantic	Frontal medial cortex	MedFC	0, 43, −19
	Left/right superior frontal g.	SFG	−14, 19, 56/15, 18, 57
	Left/right middle frontal g.	MidFG	−38, 18, 42/39, 19, 43
	Left/right frontal orbital cortex	Forb	−30, 24, −17/29, 23, −16
	eft/right inferior frontal g. pars triangularis	IFGtri	−50, 28, 9/52, 28, 8
	Left/right inferior frontal g. pars opercularis	IFGoper	−51, 15, 15/52, 15, 16
	Left/right temporal pole	TP	−40, 11, −30/41, 13, −30
	Left/right superior temporal g., anterior	aSTG	−56, −4, −8/58, −1, −10
	Left/right superior temporal g., posterior	pSTG	−62, −29, 4/61, −24, 2
	Left/right middle temporal g., anterior	aMTG	−57, −4, −22/58, −2, −25
	Left/right middle temporal g., posterior	pMTG	−61, −27, −11/61, −23, −12
	Left/right middle temporal g., temporooccipital	toMTG	−58, −53, 1/58, −49, 2
	Left/right inferior temporal g., anterior	aITG	−48, −5, −39/46, −2, −41
	Left/right inferior temporal g., posterior	pITG	−53, −28, −26/53, −23, −28
	Left/right inferior temporal g., temporooccipital	toITG	−52, −53, −17/54, −50, −17
	Left/right fusiform g., anterior	aTFusC	−32, −4, −42/31, −3, −42
	Left/right fusiform g., posterior	pTFusC	−36, −30, −25/36, −24, −28
	Left/right fusiform g., temporooccipital	TOFusC	−33, −54, −16/35, −50, −17
	Left/right parahippocampal g., anterior	aPaHC	−22, −9, −30/22, −8, −30
	Left/right parahippocampal g., posterior	pPaHC	−22, −32, −17/23, −31, −17
	Left/right supramarginal g., anterior	aSMG	−57, −33, 37/58, −27, 38
	Left/right supramarginal g., posterior	pSMG	−55, −46, 33/55, −40, 34
	Left/right angular g.	AG	−50, −56, 30/52, −52, 32
	Posterior cingulate	PC	1, −37, 30
	Precuneus	Precuneus	1, −59, 38
DAN	Left/right precentral g.	PreCG	−29, −9, 54/29, −9, 54
	Left/right supramarginal g., anterior	aSMG	−44, −39, 45/41, −39, 4
	Left/right lateral occipital cortex, superior	sLOC	−26, −66, 48/26, −66, 48
	Left/right lateral occipital cortex, inferior	iLOC	−50, −66, −6/53, −63, −6
ECN	Left/right paracingulate g.	PaCiG	0, 24, 46
	Left/right frontal pole	FP	−44, 45, 0/44, 45, 0
	Left/right angular g.	AG	−50, −51, 45/50, −51, 45
Salience	Anterior cingulate	AC	0, 21, 36
	Left/right frontal pole	FP	−35, 45, 30/32, 45, 30
	Left/right insular cortex	IC	−41, 3, 6/41, 3, 6
	Left/right supramarginal g., posterior	pSMG	−62, −45, 30/62, −45, 30
SMN	Left/right precentral g.	PreCG	0, −21, 48
	Left/right postcentral g.	PostCG	−39, −26, 51/38, −26, 48
Visual	Left/right intracalcarine cortex	ICC	−7, −83, 2/7, −83, 2
Auditory	Left/right planum temporale	PT	−62, −30, 12/59, −27, 15

*Regions and MNI coordinates for the default mode network are from the standard DMN atlas in the CONN toolbox. Regions for the semantic network were obtained by name from reviews/meta-analyses and corresponding MNI coordinates were obtained from the CONN toolbox. MNI coordinates for the dorsal attention, executive control, salience, sensorimotor, visual, and auditory networks were obtained from Raichle (2011) and matched to corresponding regions from the Harvard-Oxford atlas in the CONN toolbox*.

Once the RSNs were defined, a first-level analysis using a weighted General Linear Model (GLM) with bivariate ROI-to-ROI correlation was performed for each participant for each network. ANOVAs were run at the second level to determine the main effect of rest on network organization for each group and the comparison between groups. Additional analyses used aphasia severity and lesion size as covariates of interest to determine whether and how these factors play a role in network connectivity. Functional connectivity for each contrast was corrected at a connection-level threshold of FDR *p* < 0.05.

## Results

Results for each network will be presented separately. First the network connectivity for each group (PWA and NHA) will be presented, and then the group differences in network connectivity will be presented. Results for analyses in which the effects of aphasia severity and lesion size are explored will then be presented.

### Default mode network (DMN)

**Figure 2:**
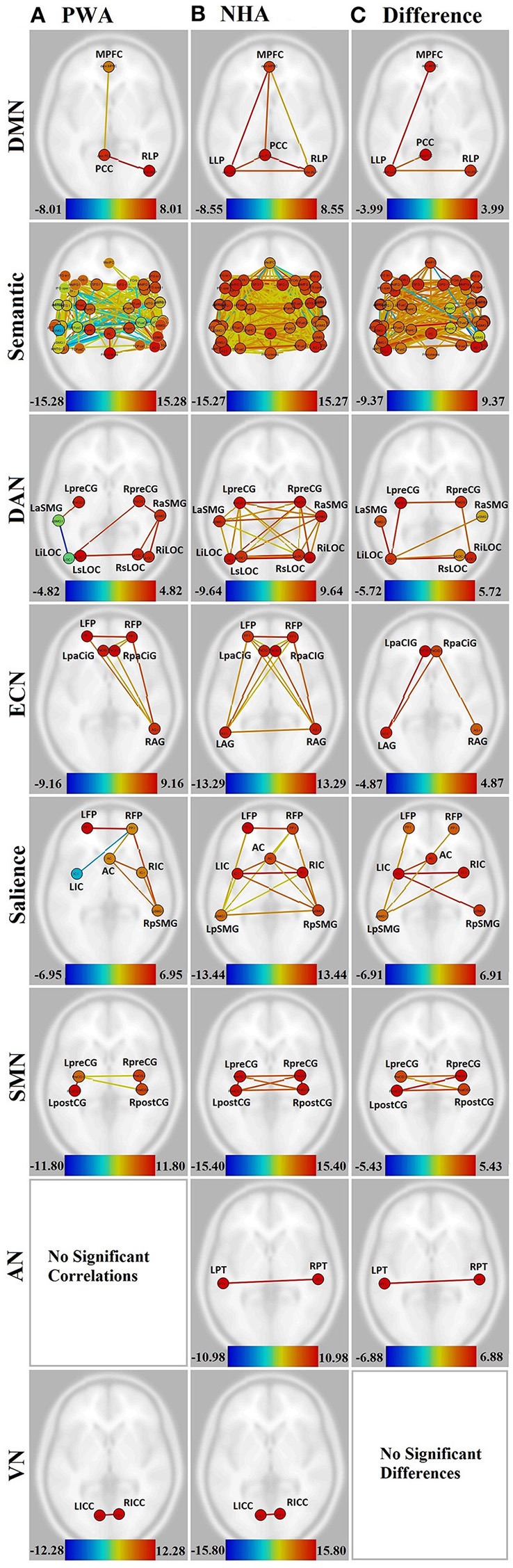
**Visualization of all networks probed across groups and contrasts**. Figures presented following neurological convention with left visualized on the left. **(A)** Persons with aphasia (PWA); **(B)** Neurologically healthy adults (NHA); **(C)** Difference in connectivity between groups. For **(A,B)**, warm colors represent statistically significant positive correlations, cool colors represent statistically significant negative correlations (anticorrelations). For **(C)**, warm colors represent statistically greater correlations for NHA, cool colors represent statistically greater correlations for PWA. See Supplementary Tables 1, 2 for exact *T*-values and FDR *p*-values.

PWA showed significant connections within the DMN from right lateral parietal (RLP) to posterior cingulate cortex (PCC), and from medial prefrontal cortex (MPFC) to PCC. In contrast, NHA showed significant correlations among all regions in the DMN. See Figures 2A,B and Supplementary Table 1.

When directly compared, NHA showed significantly greater connection strength within the DMN than PWA, including connections between left lateral parietal (LLP) and MPFC, PCC, and RLP. See Figure 2C and Supplementary Table 3.

### Semantic network

While both PWA and NHA showed many connections within the semantic network during rest, the number of significant positive connections in the NHA network (684) was three and a half times the number of significant positive connections in the PWA network (198). Additionally, the NHA network was balanced across the hemispheres with 44% cross-hemispheric, 30% left-lateralized, and 26% right-lateralized connections. Whereas, the PWA network was right-lateralized with 22% cross-hemispheric, 29% left-lateralized, and 48% right-lateralized connections. To confirm lateralization, a laterality index (LI) was calculated by subtracting the number of right-hemisphere connections from the number of left-hemisphere connections and dividing by the total number of connections, resulting in a laterality index of −0.24, which passes the commonly used threshold of ±0.20 (Seghier, 2008). See Figures 2A,B, Supplementary Figures 1A,B, and Supplementary Table 2.

Interestingly, both NHA and PWA exhibited anticorrelations in the semantic network; however, PWA produced over seven times more anticorrelations in the semantic network than NHA (52 vs. 7). Furthermore, while NHA exhibited only one cross-hemispheric anticorrelation and significant right-laterization (LI = −0.33), 79% of PWA anticorrelations were cross-hemispheric and the remaining connections were significantly left-lateralized (LI = 0.64). See Figures 2A,B, Supplementary Figures 1A,B, and Supplementary Table 2.

For direct comparisons, only connections that were significant for either group separately were retained to avoid misinterpretation of differences of network connectivity strength. NHA showed significantly greater connection strength than PWA for 173 connections within the semantic network. While the majority were cross-hemispheric (104), there were many more left-lateralized (49) than right-lateralized (19) connections that were stronger for NHA than PWA. This was confirmed with a laterality index of 0.44. PWA showed significantly greater connection strength than NHA for 23 connections. Two were cross-hemispheric, 10 were left-lateralized, and 11 were right-lateralized. See Figure 2C, Supplementary Figure 1C, and Supplementary Table 4.

### Dorsal attention network (DAN)

Significant connections within the DAN for PWA involved mainly right posterior regions (LI = −0.60). Again, PWA exhibited an anticorrelation between left anterior supramarginal gyrus (aSMG) and left inferior lateral occipital cortex (iLOC). In contrast, NHA showed significant correlations among all regions in the DAN, with no anticorrelations. See Figures 2A,B and Supplementary Table 1.

When directly compared, NHA showed significantly greater connection strength than PWA for seven connections within the DAN, four of which were cross-hemsipheric. The remaining connection differences were left-lateralized (LI = 0.33). See Figure 2C and Supplementary Table 1.

### Executive control network (ECN)

PWA showed eight significant connections within the ECN, including cross-hemispheric prefrontal connections and connections between prefrontal cortex and right angular gyrus (AG). In contrast, NHA showed 15 significant correlations among all regions in the ECN. See Figures 2A,B and Supplementary Table 1.

When directly compared, NHA showed significantly greater connection strength than PWA within the ECN, including connections between left AG and bilateral paracingulate (PaCiG), and between right AG and right PaCiG. See Figure 2C and Supplementary Table 1.

### Salience network

PWA showed six significant positive connections within the salience network, two of which were cross-hemispheric, with the remaining four being right-lateralized. Again, PWA exhibited an anticorrelation between right frontal pole (FP) and left insular cortex (IC). In contrast, NHA showed 14 significant correlations among all regions in the salience network. Missing connections included bilateral within- and cross-hemispheric FP-IC, and between left FP and AC. See Figures 2A,B and Supplementary Table 1.

When directly compared, NHA showed significantly greater connection strength within the salience network than PWA, including connections between left IC and right IC, pSMG, and AC, and between left pSMG and right IC and bilateral FP. Again, greater connection strength was within cross-hemispheric and left-lateralized connections. See Figure 2C and Supplementary Table 1.

### Sensorimotor network (SMN)

In the SMN, PWA were missing cross-hemispheric connections between left and right postcentral gyrus (postCG), and from left postCG to right precentral gyrus (preCG). In contrast, NHA showed significant correlations among all regions within the SMN. See Figures 2A,B and Supplementary Table 1.

When directly compared, NHA showed significantly greater connection strength within the SMN than PWA, including all cross-hemispheric connections. See Figure 2C and Supplementary Table 1.

### Auditory network

The connection between the two nodes of the auditory network [L-R planum temporale (PT)] was not significant for PWA, but was significant for NHA. See Figures 2A,B and Supplementary Table 1. When directly compared, NHA showed significantly greater connection strength within the auditory network than PWA. See Figure 2C and Supplementary Table 1. Note that although PT, rather than Heshl's gyrus, was the region in the HO atlas that coincided with the MNI coordinates used to define the auditory network, it overlaps with BA41 (OBART: Online Brain Reconciliation Tool; Bohland et al., 2009). As a check, analyses were also run on Heshl's gyrus and the same results were found.

### Visual network

Both PWA and NHA showed a significant connection between the two nodes of the visual network [L-R intracalcarine cortex (ICC)]. See Figures 2A,B and Supplementary Table 1.

When directly compared, no differences between groups emerged. See Figure 2C and Supplementary Table 1.

### Effect of aphasia severity and lesion size

**Figure 3:**
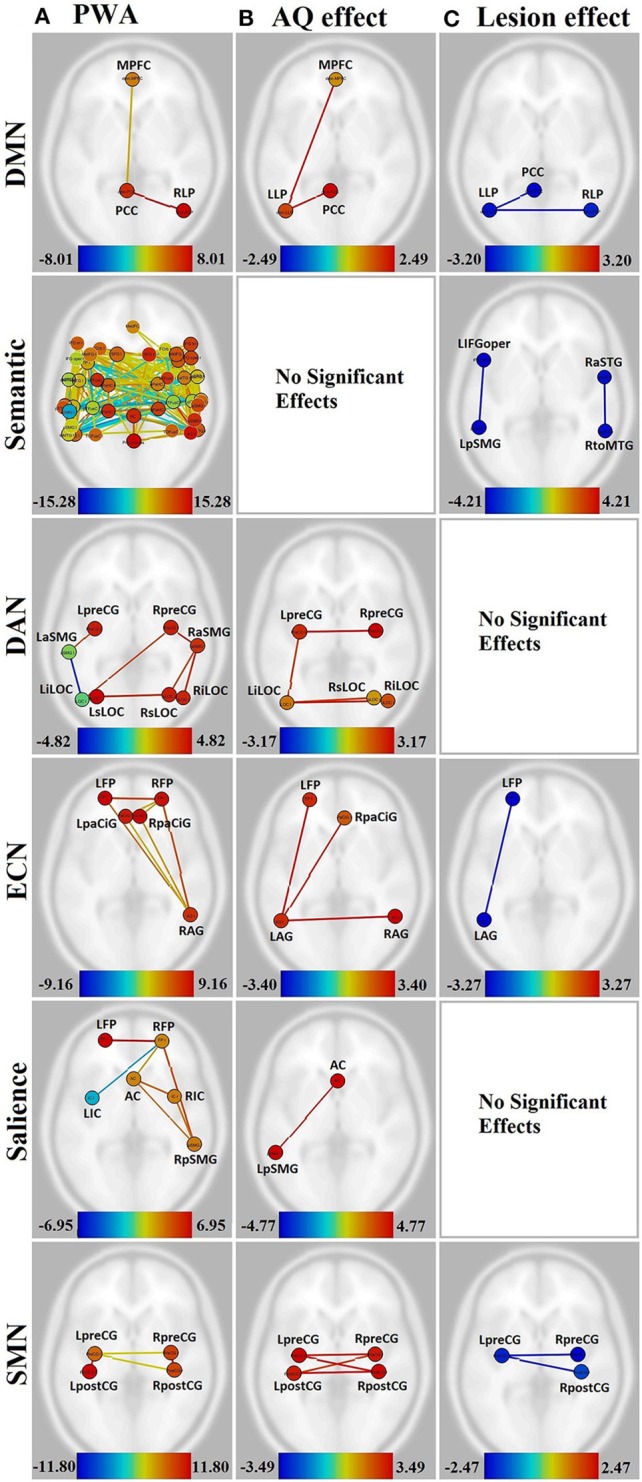
**Visualization of the effect of aphasia severity and lesion size on connectivity within affected networks for persons with aphasia**. Figures presented following neurological convention with left visualized on the left. **(A)** Unmodulated networks in persons with aphasia (PWA); **(B)** Effect of AQ (aphasia severity); **(C)** Effect of lesion. For **(A)**, warm colors represent statistically significant positive correlations, cool colors represent statistically significant negative correlations (anticorrelations). For **(B,C)**, warm colors represent statistically significant positive relationships between connectivity and demographic measure, cool colors represent statistically significant negative relationships between connectivity and demographic measure.

Additional analyses were performed to determine the effect of lesion size and aphasia severity on the connectivity within the group of PWA, and also on the difference in connectivity between groups. The WAB Aphasia Quotient (AQ) was normalized across the PWA group sample for use as a measure of aphasia severity. The normalized AQ was entered into an ANCOVA and weighted as a main effect (i.e., NHA, PWA, and AQ were weighted as 0, 0, 1, respectively). For the lesion analysis, the total lesion size was normalized across the PWA group sample and entered into a separate ANCOVA, and weighted as a main effect (i.e., NHA, PWA, and lesion size were weighted as 0, 0, 1, respectively). This analysis revealed increased connectivity with decreasing aphasia severity and decreased connectivity with increasing lesion size in several networks. See Figures 3B,C. To further probe how this might affect group differences, a comparison between groups (in which NHA and PWA were weighted at 1 and −1, respectively, with the covariate weighted as 0) was run only with connections that were significantly affected by lesion or AQ, using lesion or AQ as a covariate, respectively.

First, this group of PWA was quite heterogenous in lesion size and AQ (see Table 1). Although, negatively correlated (*r* = −0.64, *p* < 0.01), note that the relationship is not absolute (e.g., the lowest AQ did not represent the largest lesion). The highest lesion overlap among PWA was in parts of the left superior corona radiata, internal capsule, and superior longitudinal fasciculus. See Table 3 for exact percent spared tissue for each ROIs. Notably, PWA02 and PWA03 had the largest lesions and therefore had the most ROIs that were <50% spared. Importantly, of the 40 ROIs (across networks), only toMTG was <50% spared in more than two of the seven PWA, 14 ROIs were <50% spared in two PWA, 9 ROIs were <50% spared in one PWA, and 16 ROIs were >50% spared (and often 90–100% spared) by all seven PWA. Thus, the vast majority of ROIs were at least 50% spared in the majority of PWA.

**Table 3:** **Percent spared tissue in LH ROIs within each network for each PWA**.

	**PWA01 (%)**	**PWA02 (%)**	**PWA03 (%)**	**PWA04 (%)**	**PWA05 (%)**	**PWA06 (%)**	**PWA07 (%)**
**DMN**							
LLP	100	*2*	*17*	77	100	100	100
PCC	100	*42*	100	93	100	77	100
MPFC	100	100	100	100	100	100	100
RLP	100	100	100	100	100	100	100
**SEMANTIC**							
AG	72	*0*	*3*	60	100	100	100
aITG	100	94	81	100	100	100	100
aMTG	100	*25*	*24*	100	100	100	100
aPaHC	100	100	75	100	100	100	100
aSMG	100	*5*	*8*	89	100	98	100
aSTG	100	*13*	*0*	100	100	100	100
aTFusC	100	100	74	100	100	100	100
Forb	100	76	*36*	100	100	100	100
IFGop	100	53	*0*	100	100	53	100
IFGtri	100	85	*6*	100	100	94	100
MedFC	100	100	100	100	100	100	100
MidFG	100	71	*41*	93	100	*27*	100
PC	100	73	100	90	100	97	100
pITG	100	96	66	96	100	100	100
pMTG	97	*39*	*11*	84	100	100	100
pPaHC	100	100	100	100	100	100	100
precuneus	100	79	98	93	100	92	100
pSMG	93	*1*	*7*	74	100	100	100
pSTG	100	*22*	*0*	80	100	100	100
pTFusC	100	96	97	100	100	100	100
SFG	100	94	99	85	100	*38*	100
TOFusC	100	100	100	89	100	100	100
toITG	100	100	75	*43*	100	100	100
toMTG	51	*40*	*4*	*9*	100	100	100
TP	100	59	*22*	100	100	100	100
**DAN**							
aSMG	100	*5*	*8*	89	100	98	100
iLOC	100	99	51	*6*	100	100	100
preCG	100	51	*43*	91	100	51	100
sLOC	98	*32*	*48*	68	100	98	100
**ECN**							
AG	72	*0*	*3*	60	100	100	100
FP	100	99	89	100	100	100	100
PaCiG	100	92	100	100	100	79	100
**SALIENCE**							
AC	77	100	96	100	100	76	100
FP	100	99	89	100	100	100	100
IC	81	*7*	*0*	100	100	100	97
pSMG	93	*1*	*7*	74	100	100	100
**SMN**							
postCG	100	*18*	*41*	95	100	82	100
preCG	100	51	*43*	91	100	51	100
**VISUAL**							
ICC	100	100	100	100	100	100	100
**AUDITORY**							
HG	100	*0*	*0*	100	100	100	100
PT	100	*2*	*0*	72	100	100	100

*Regions with <50% spared tissue are italicized and underlined*.

In the DMN, lower aphasia severity (i.e., higher AQ) was associated with higher connectivity for left hemisphere connections, including LLP-MPFC [*T*_(15)_ = 2.49, *p* < 0.05] and LLP-PCC [*T*_(15)_ = 2.41, *p* < 0.05]. Both of these connections showed higher connectivity for NHA than PWA with increasing severity (*p* < 0.01 for both). Larger lesion size was associated with decreasing connectivity in the LLP-PCC [*T*_(15)_ = −2.90, *p* < 0.05] and LLP-RLP [*T*_(15)_ = −3.20, *p* < 0.05] connections; however, lesion size did not significantly affect the difference between groups for these connections.

In the semantic network, aphasia severity did not modulate connectivity, although larger lesion size was associated with decreasing connectivity in L-L IFGoper-pSMG [*T*_(15)_ = −4.10, *p* < 0.05] and R-R aSTG-toMTG [*T*_(15)_ = −4.21, *p* < 0.05]. Of these, the R-R aSTG-toMTG connection showed higher connectivity for PWA than NHA with decreasing lesion size (*p* < 0.05).

In the DAN, higher AQ was associated with stronger left- and cross-hemispheric connections, including L-R preCG [*T*_(15)_ = 3.17, *p* < 0.05], L-R iLOC [*T*_(15)_ = 2.97, *p* < 0.05], left iLOC-right sLOC [*T*_(15)_ = 2.64, *p* < 0.05], and L-L preCG-iLOC [*T*_(15)_ = 2.59, *p* < 0.05]. All of these connections showed higher connectivity for NHA than PWA with increasing severity (*p* < 0.01 for all). No significant effects were found for lesion size.

In the ECN, higher AQ was associated with stronger left- and cross-hemispheric connections, including L-L AG-FP [*T*_(15)_ = 3.40, *p* < 0.05], L-R AG [*T*_(15)_ = 3.33, *p* < 0.05], and left AG-right PaCiG [*T*_(15)_ = 3.12, *p* < 0.05]. All of these connections showed higher connectivity for NHA than PWA with increasing severity (*p* < 0.001 for all). Larger lesion size was associated with decreasing connectivity in L-L AG-FP [*T*_(15)_ = −3.27, *p* < 0.05]; however, lesion size did not significantly affect the difference between groups for this connection.

In the salience network, higher AQ was associated with stronger connectivity between the AC and left pSMG [*T*_(15)_ = 4.77, *p* < 0.01], and this connection showed higher connectivity for NHA than PWA with increasing severity (*p* < 0.001). No significant effects were found for lesion size.

In the SMN, higher AQ was associated with stronger cross-hemispheric connections, including L-R postCG [*T*_(15)_ = 3.49, *p* < 0.01], L-R preCG [*T*_(15)_ = 3.17, *p* < 0.05], left preCG-right postCG [*T*_(15)_ = 3.43, *p* < 0.01], and left postCG-right preCG [*T*_(15)_ = 2.83, *p* < 0.05]. All of these connections showed higher connectivity for NHA than PWA with increasing severity (*p* < 0.001 for all). Larger lesion size was associated with decreasing connectivity between L-R preCG [*T*_(15)_ = −2.47, *p* < 0.05] and left preCG-right post-CG [*T*_(15)_ = −2.40, *p* < 0.05]. Of these, the L-R preCG connection showed higher connectivity for NHA than PWA with increasing lesion size (*p* < 0.05).

In the visual and auditory networks, there were no significant effects of aphasia severity or lesion size.

## Discussion

In this data set, the RSNs—including a semantic network—of NHA aged 47–74 contained all expected regions and were densely connected. As hypothesized, PWA showed hypoconnectivity in both traditional RSNs and the additional semantic network. The only network that did not show hypoconnectivity in PWA was the visual network, which did not contain any regions that were lesioned in this specific group of PWA and are not normally lesioned in this population. In other words, for PWA, within every network probed except the visual network, not only were regions within a network not always connected with each other, but some left-hemisphere regions were not connected to the network at all. When directly compared, the difference between “normal” connectivity for these networks and that of PWA was confirmed, with the bulk of differences tending to be cross-hemispheric or left-lateralized.

When lesion size was considered as a main effect, some of the missing or disconnected regions were accounted for, but this was not always the case. As can be seen in Figure 3C vs. Figure 3A, lesion size had no significant effect on the dorsal attention and salience networks, although these had missing or hypoconnected left hemisphere regions.

Importantly, many of the regions that were missing or hypoconnected within PWA networks were at least 50% spared in five of the seven PWA, and some were substantially spared in all seven PWA. For example, MPFC and RLP are 100% spared in all seven PWA, but the expected connection between these is not significant in this group. See Table 3 for percent spared tissue in each region for each PWA, organized by network. MPFC is considered to be a hub of the DMN and is important for self-referential processing and may be involved in initiation of internal conceptual processing (Binder et al., 1999; Buckner et al., 2008). Thus, the hypoconnectivity of this node within the DMN of PWA, even when it is 100% spared is troubling and may be clinically relevant. Notably, Eldaief et al. (2016) found that although the DMN was largely unchanged when MPFC was focally lesioned, couplings between the DMN and other networks were affected, suggesting that this region is important for integration of information among networks.

Similarly, within the DAN, left iLOC is nearly 100% spared in five PWA, 51% spared in one PWA, and 6% spared in one PWA. Left iLOC has a significant connection with right preCG in PWA, but no other region. NHA show higher connectivity than PWA between left iLOC and right iLOC (100% spared in PWA), right sLOC (100% spared in PWA), right aSMG (100% spared in PWA), left aSMG (89–100% spared in five PWA, 5–8% spared in two PWA), and left preCG (43–100% spared in PWA; See Table 3). These examples suggest that the effect of lesion may be more far-reaching than simply removing a node from a network if that node is completely lesioned.

This is not altogether unexpected, as it is known that stroke can affect regions distant to the frank infarct (Seitz et al., 1999), and as mentioned in the introduction, focal lesions can impact global network connectivity patterns (Gratton et al., 2012). Further, recent work has shown the relationship between white matter pathways and stroke recovery (e.g., Geva et al., 2015). In this data set, the highest amount of lesion overlap was in white matter (See Figure 1). Thus, white matter damage in this patient sample is most likely contributing to the observed network (dis-)organization. Other factors, such as the process of re-organization during stroke recovery, may also affect the effect of lesion on network connectivity. The PWA in this study ranged from seven to 134 months post stroke, ranged in age from 47 to 74, and ranged in lesion size from 0.33 to 255.95 cc. All of these stroke factors—and many demographic variables not mentioned here, including education level—can contribute to the individual variability of recovery processes and should be expected to affect the re-organization of network structure. Future work should address individual variability and possible factors that may affect network re-organization during stroke recovery.

Although, aphasia severity and lesion size are often related (and negatively correlated in this group), there is not a mirror-image effect on connectivity. In this data set, aphasia severity seemed to have more of an effect on network connectivity than lesion size. Increasing AQ (decreasing severity) was associated with increasing strength of left-hemisphere and cross-hemispheric connections within every network except the semantic and auditory networks. This seems counterintuitive, since AQ is a language measure that most certainly relies on the semantic and auditory networks. However, perhaps it should not be surprising that increasing aphasia severity can be linked with decreasing connectivity (i.e., more hypoconnectivity) in the default mode, dorsal attention, executive control, salience, and sensorimotor networks. As mentioned in the introduction, other cognitive functions besides language are gaining attention in the clinical aphasiology literature. This may, in large part, be due to the fact that while isolated language deficits do exist, and may help researchers to pinpoint certain language functions, the majority of clients seen by speech language pathologists have a constellation of deficits, not limited to language. The results of this study support the notion that aphasia severity may be related to disorganization or hypoconnectivity in networks that support extralinguistic cognition. This has implications for recovery of function in aphasia, not only for specific non-linguistic cognitive processes that may be impaired, but also for language due to the interaction of language and other cognitive functions. Future work should test the hypothesis that aphasia severity is linked with general cognitive health as measured by the integrity of RSNs, and additionally explore the links between a variety of performance measures and patterns of connectivity at rest.

In this study, an additional semantic network was included based on the ubiquitous presence of word-finding deficits in aphasia. Regions were included in the semantic network based upon reviews of semantic processing (Binder et al., 2009; Price, 2012). The group of NHA exhibited an extremely dense and well-connected semantic network with a majority of cross-hemispheric connections. PWA, on the other hand, exhibited a relatively sparsely connected semantic network that was slightly right-lateralized. When directly compared, NHA exhibited stronger cross- and left-hemispheric connections than PWA. This suggests that left hemisphere lesions reduce communication both within the lesioned hemisphere and between hemispheres. PWA showed some stronger intra-hemispheric connections than NHA within the semantic network, mainly in inferior temporal regions, which are largely spared by the lesions. This may indicate compensatory mechanisms, though no significant correlations with aphasia severity were found. Many of the regions that showed stronger connections for NHA were in the temporal lobe, especially temporo-occipital middle temporal gyrus, where four of the seven PWA exhibited substantial damage. However, disruptions to the semantic network were not only occurring in regions with frank lesions. For example, NHA showed stronger cross-hemispheric connectivity than PWA in inferior temporal gyrus (anterior, posterior, and temporo-occipital) homologs, although this region was largely spared in PWA. This again points to the notion that regions spared by the lesion can still have altered connectivity patterns.

The temporal lobe has long been considered an important region for semantic processing. Thus, the idea that regions of the temporal lobe can be spared, but dis- or hypo-connected within the semantic network may help to more fully understand the underlying nature of word-finding deficits in aphasia. Again, these preliminary results require replication and extension with a larger, more diverse group of PWA to make any firm conclusions.

Of note was the existence of anticorrelations in the semantic, dorsal attention, and salience networks for PWA. Anticorrelations have been found between networks during rest, signaling the difference between task-positive networks—regions involved in goal-directed tasks—and task-negative networks—regions that are more active during rest than goal-directed tasks (Fox et al., 2005). Thus, anticorrelations help delineate separable functional networks and have been shown to be reliable and not simply an artifact of global signal regression (Chai et al., 2012). This study used CompCor (Behzadi et al., 2007), which is more robust for anticorrelations (Whitfield-Gabrieli and Nieto-Castanon, 2012). Importantly, the anticorrelations observed in this study were not *between*-network, but *within*-network. This indicates that the RSNs in PWA are not only hypoconnected, but may also be abnormally organized. Abnormal network organization evidenced by the unexpected presence/absence of anticorrelations has been observed in other patient groups, including schizophrenia, bipolar disorder, and Alzheimer's disease (Wang et al., 2007; Chai et al., 2011).

It may not be surprising that the anticorrelations involved left-hemisphere regions that were <50% spared, as the presence of a lesion may necessitate network re-organization around the lesioned tissue. However, in the DAN, one of the regions (left iLOC) was more than 50% spared in all but one PWA, and in the salience network, one of the regions (right FP) was 100% spared in all PWA. In the semantic network, there appear to be a few regions that are more anticorrelated than others. Regions with more than five anticorrelations that were <50% spared in more than one PWA included left aMTG, aSMG, and toMTG. However, the majority of regions with more than five anticorrelations were not heavily damaged regions: left aITG was more than 81% spared in all PWA; left pITG was more than 66% spared in all PWA; right pITG was more than 100% spared in all PWA; and left TP was more than 59% spared in all but one PWA. The region with the most anticorrelations (by nearly double) was posterior temporal fusiform cortex bilaterally, which was at least 96% spared in all PWA. This result is surprising because not only was this region spared, it was involved bilaterally (i.e., left and right each had 10 anticorrelations), and it is located in ventral temporal cortex, which has been shown to be involved in visual object processing and appears to have category specificity (Grill-Spector and Weiner, 2014). This suggests abnormal organization of the semantic network in this group of PWA, and also underscores the notion that a lesion can have non-localized effects. Importantly, this region showed no anticorrelations in the NHA, further supporting the presence of abnormal organization of networks in PWA. Rather, the region with the most anticorrelations in the NHA was medial prefrontal cortex. Perhaps this region is more associated with the DMN than the semantic network, though previous work has suggested that this is one of the regions that overlaps between the two networks (Binder et al., 2009).

It is important to note some limitations of this study. First, the sample size for each group was small, especially the PWA. However, the results were as predicted and supported the hypotheses. Also, some interesting, but logical patterns emerged that introduced new questions to be tested in a larger group of PWA. Second, the data were acquired on two different scanners. However, the protocols were matched as closely as possible and recent work has shown the impact of scanner differences to be minimal (Forsyth et al., 2014). Because smaller sample sizes may be more susceptible to scanner differences due to individual variability, as a check, the NHA data collected at Penn State were compared against NHA data from the Human Connectome Project, approximately matched on group size (*N* = 10) and age (*M* = 60). There were no differences in any RSN except the semantic network, in which only 3 connections (0.1% of possible) were different, and the ECN, in which only 1 connection (3% of possible) were different. This lack of difference is impressive considering the small sample sizes and inherent individual variability, as well as protocol and scanner differences, and supports the validity of the comparison between groups made in this study.

In conclusion, RSNs, especially the DMN, have begun to be thought of as an indicator of general cognitive health. This is partly due to the successful use of rs-fMRI to differentiate dementia from mild cognitive impairment, but is also gaining popularity in other clinical realms, such as TBI, stroke, schizophrenia, and autism. However, the usefulness of this tool to measure general cognitive health in aphasia is currently unknown, as is the utility of measuring general cognitive health in aphasia. Though small in sample size, the results of this pilot work positively contribute to the limited evidence supporting the use of rs-fMRI in aphasia. These data suggest that (a) PWA exhibit hypoconnectivity in the semantic network and all RSNs except the visual network, (b) connectivity decreases with increasing lesion size, and (c) connectivity increases with decreasing aphasia severity. Thus, general cognitive health appears to be affected in aphasia, and rs-fMRI appears to be a sensitive tool to measure general cognitive health (and the health of the semantic system) in aphasia. The use of rs-fMRI in aphasia research has the potential to improve aphasia therapy by furthering the understanding of lesion effects on the cognitive system as a whole, which can guide treatment target selection and promotion of favorable neural reorganization for optimal recovery of function. Future work will replicate these findings in a larger group of PWA and examine the effect of treatment on rs-fMRI activity and network connectivity.

## Ethics statement

Boston University Institutional Review Board; Penn State University Institutional Review Board. Each participant reviewed and signed a consent form approved by the university's IRB. Each PWA was consented by a student clinician with a Master's degree in Speech-Language Pathology or a licensed Speech-Language Pathologist, who was trained to provide comprehension support if needed.

## Author contributions

CS designed the study, carried out the acquisition, analysis, and interpretation of the data, drafted the manuscript, and is solely accountable for all aspects of the work.

## Funding

This work was funded by an F31 Ruth L. Kirschstein National Research Service Award awarded to the author by the National Institutes of Health, National Institutes on Deafness and other Communication Disorders (1F31DC011220-01A1), and a New Centuries Scholars Grant awarded to the author by the American Speech-Language Hearing Foundation.

### Conflict of interest statement

The author declares that the research was conducted in the absence of any commercial or financial relationships that could be construed as a potential conflict of interest.

## Supplementary material

Supplementary Table 1 **Statistically significant connections in resting state networks for both groups**.

Supplementary Table 2 **Significant connections in the semantic network for both groups**.

Supplementary Table 3 **Connection differences between groups in resting state networks**.

Supplementary Table 4 **Connection differences between groups in the semantic network**.

Supplementary Figure 1 **Sagittal and coronal views of semantic network. (A)** Persons with aphasia (PWA); **(B)** Neurologically healthy adults (NHA); **(C)** Difference in connectivity between groups. For **(A,B)**, warm colors represent statistically significant positive correlations, cool colors represent statistically significant negative correlations (anticorrelations). For **(C)**, warm colors represent statistically greater correlations for NHA, cool colors represent statistically greater correlations for PWA. See Supplementary Tables 1, 2 for exact *T*-values and FDR *p*-values.
