# Supplementary material for: Hypoconnectivity of Resting-State Networks in Persons with Aphasia Compared with Healthy Age-Matched Adults
Source: Front Hum Neurosci. 2017 Feb 28;11:91. doi: 10.3389/fnhum.2017.00091 (PMC5329062; doi:10.3389/fnhum.2017.00091)
Supplement: Supplementary Table 1 — Statistically significant connections in resting state networks for both groups. [file Table1.pdf]

Supplementary Table 1

Statistically Significant Connections in Resting State Networks for Both Groups

Neurologically Healthy Adults			Persons with Aphasia		
Connection	T(16)	p-FDR	Connection	T(16)	p-FDR
Default Mode Network					
MPFC-LLP	8.55	0.000	RLP-PCC	8.01	0.000
PCC-RLP	7.57	0.000	PCC-MPFC	2.41	0.043
RLP-LLP	5.75	0.000			
PCC-LLP	5.67	0.000			
PCC-MPFC	5.30	0.000			
RLP-MPFC	2.83	0.012			
Dorsal Attention Network					
PreCGl-PreCGr	9.64	0.000	sLOCr-iLOCr	4.82	0.001
iLOCl-iLOCr	9.33	0.000	aSMGr-PreCGr	4.39	0.003
sLOCl-sLOCr	9.28	0.000	aSMGr-iLOCr	3.87	0.005
PreCGr-iLOCl	7.13	0.000	aSMGr-sLOCr	3.41	0.008
iLOCr-PreCGr	6.36	0.000	sLOCl-sLOCr	3.86	0.010
aSMGr-aSMGl	6.24	0.000	iLOCl-PreCGr	3.53	0.010
iLOCl-sLOCr	5.97	0.000	PreCGl-aSMGl	3.26	0.034
iLOCr-sLOCr	5.70	0.000	Anticorrelations		
iLOCl-aSMGr	5.53	0.000	iLOCl-aSMGl	-3.87	0.010
PreCGr-aSMGr	5.09	0.000			
aSMGr-PreCGl	4.74	0.000			
aSMGr-sLOCr	4.64	0.000			
iLOCl-PreCGl	4.29	0.001			
iLOCr-aSMGr	4.29	0.001			
iLOCl-sLOCl	4.09	0.001			
iLOCr-PreCGl	3.96	0.002			
iLOCr-sLOCl	3.59	0.003			
PreCGr-aSMGl	3.49	0.004			
PreCGl-aSMGl	3.45	0.005			
PreCGl-sLOCl	3.31	0.005			
aSMGl-iLOCl	3.30	0.008			
sLOCr-PreCGl	2.85	0.014			
PreCGr-sLOCr	2.84	0.014			
sLOCl-PreCGr	2.47	0.035			
sLOCr-aSMGl	2.21	0.042			
Executive Control Network					
PaCiGl-PaCiGr	13.29	0.000	PaCiGr-PaCiGl	9.16	0.000
FPl-FPr	10.27	0.000	FPl-FPr	6.95	0.000
PaCiGr-AGr	8.60	0.000	FPr-AGr	6.23	0.000
PaCiGl-AGl	8.39	0.000	AGr-FPl	4.91	0.000
AGr-FPr	7.97	0.000	AGr-PaCiGr	3.53	0.005
PaCiGr-FPr	6.51	0.000	PaCiGr-FPr	3.49	0.005
AGl-AGr	5.97	0.000	FPr-PaCiGl	2.91	0.013

PaCiGl-FPl	5.49	0.000	AGr-PaCiGl	2.88	0.014
AGl-PaCiGr	5.46	0.000			
AGl-FPl	5.19	0.000			
FPl-AGr	4.94	0.000			
PaCiGl-AGr	4.25	0.001			
PaCiGl-FPr	3.51	0.003			
PaCiGr-FPl	3.24	0.005			
FPr-AGl	3.17	0.006			
<u>Salience Network</u>					
ICr-ICl	13.44	0.000	FPr-FPl	6.95	0.000
FPl-FPr	10.27	0.000	pSMGr-FPr	4.85	0.001
ICl-pSMGr	8.01	0.000	AC-ICr	4.03	0.005
ICr-AC	7.94	0.000	pSMGr-AC	3.83	0.005
ICr-pSMGr	7.88	0.000	ICr-pSMGr	3.55	0.008
ICl-AC	7.75	0.000	FPr-AC	2.55	0.041
pSMGr-FPr	6.12	0.000	<u>Anticorrelations</u>		
pSMGr-pSMGl	5.61	0.000	FPr-ICl	-2.43	0.041
pSMGr-AC	5.18	0.000			
FPl-pSMGl	4.41	0.001			
pSMGl-FPr	3.48	0.006			
ICr-pSMGl	3.06	0.011			
AC-FPr	3.00	0.013			
ICl-pSMGl	2.37	0.046			
<u>Sensorimotor Network</u>					
PostCGr-PreCGr	15.40	0.000	PostCGr-PreCGr	11.80	0.000
PreCGl-PostCGl	13.17	0.000	PreCGl-PostCGl	9.95	0.000
PreCGr-PostCGl	10.16	0.000	PreCGl-PostCGr	2.55	0.029
PostCGr-PostCGl	9.68	0.000	PreCGr-PreCGl	2.40	0.043
PreCGl-PreCGr	9.64	0.000			
PreCGl-PostCGr	8.90	0.000			
<u>Auditory Network</u>					
PTl-PTr	18.94	0.000	Nonsignificant		
<u>Visual Network</u>					
ICCl-ICCr	17.51	0.000	ICCl-ICCr	12.28	0.000

Note. See Table 2 for a key to region abbreviations.
