# Supplementary material for: Hypoconnectivity of Resting-State Networks in Persons with Aphasia Compared with Healthy Age-Matched Adults
Source: Front Hum Neurosci. 2017 Feb 28;11:91. doi: 10.3389/fnhum.2017.00091 (PMC5329062; doi:10.3389/fnhum.2017.00091)
Supplement: Supplementary Table 2 — Significant connections in the semantic network for both groups. [file Table2.pdf]

Supplementary Table 2

Significant Connections in the Semantic Network for Both Groups

Neurologically Healthy Adults			Persons with Aphasia		
Connection	T(16)	p-FDR	Connection	T(16)	p-FDR
MedFC-aMTGr	15.27	0.000	pITGl-pTFusCl	15.28	0.000
SFGl-SFGr	12.23	0.000	pTFusCr-toITGr	12.05	0.000
TPr-FOrbl	12.05	0.000	pITGr-pTFusCr	12.03	0.000
FOrbl-FOrbr	11.80	0.000	aITGl-toMTGl	11.49	0.000
PC-aMTGr	11.75	0.000	PC-Precuneous	9.57	0.000
aMTGl-TPl	11.18	0.000	aPaHCr-pPaHCr	9.31	0.000
SFGl-MidFGl	10.87	0.000	pITGr-toITGr	9.22	0.000
PC-pPaHCl	10.40	0.000	pTFusCl-toMTGl	8.86	0.000
TOFusCr-TOFusCl	10.28	0.000	aMTGr-TPr	8.85	0.000
aMTGr-aMTGl	10.17	0.000	aITGl-pTFusCl	8.46	0.000
aPaHCl-aTFusCl	10.07	0.000	aMTGl-TPl	8.09	0.000
pMTGr-pMTGl	9.93	0.000	SFGl-MidFGl	7.97	0.000
pTFusCl-TOFusCl	9.73	0.000	aPaHCr-TPr	7.94	0.000
SFGr-pMTGr	9.73	0.000	aITGr-pITGr	7.89	0.000
pSMGr-aSMGr	9.70	0.000	SFGl-SFGr	7.83	0.000
IFGoperr-pSMGr	9.54	0.000	pMTGl-toMTGl	7.77	0.000
aITGr-pITGr	9.54	0.000	pITGl-toMTGl	7.68	0.000
IFGoperl-MidFGl	9.51	0.000	pPaHCl-pPaHCr	7.49	0.000
pPaHCr-pPaHCl	9.45	0.000	toMTGr-AGr	7.41	0.000
MedFC-PC	9.39	0.000	pTFusCl-toITGl	7.34	0.000
PC-Precuneous	9.32	0.000	aPaHCl-aTFusCl	7.23	0.000
TPr-aPaHCr	9.15	0.000	aPaHCr-pPaHCl	7.21	0.000
toMTGr-toMTGl	8.93	0.000	pTFusCl-TPl	7.11	0.000
TPr-TPl	8.90	0.000	pITGr-pMTGr	7.05	0.000
pTFusCr-pTFusCl	8.89	0.000	pITGl-pMTGl	7.01	0.000
pSTGr-pSTGl	8.88	0.000	TPr-FOrbl	6.91	0.000
aPaHCl-aPaHCr	8.86	0.000	aPaHCl-pPaHCl	6.91	0.000
FOrbr-IFGtrir	8.86	0.000	aPaHCl-aPaHCr	6.64	0.000
MidFGr-MidFGl	8.79	0.000	pITGr-toMTGr	6.62	0.000
FOrbl-SFGl	8.79	0.000	aITGr-toITGr	6.57	0.000
MedFC-aMTGl	8.73	0.000	PC-AGr	6.55	0.000
toMTGl-IFGoperl	8.72	0.000	toMTGl-toITGl	6.49	0.000
SFGl-AGl	8.67	0.000	pMTGr-toMTGr	6.48	0.000
AGr-SFGr	8.63	0.000	pTFusCr-toMTGr	6.41	0.000
pMTGl-aMTGl	8.58	0.000	aMTGr-pMTGr	6.37	0.000
aMTGr-pMTGr	8.57	0.000	TPr-FOrbr	6.36	0.000
toMTGr-IFGoperr	8.39	0.000	MidFGr-SFGr	6.34	0.000
pSTGr-TPr	8.34	0.000	pITGl-toITGl	6.27	0.000
TPr-FOrbr	8.33	0.000	toMTGr-toITGr	6.24	0.000
aMTGl-SFGl	8.31	0.000	pMTGr-toITGr	6.21	0.000
PC-pMTGr	8.30	0.000	aITGl-toITGl	6.10	0.000

pPaHCl-Precuneous	8.21	0.000	pITGr-MidFGl	6.05	0.000
aMTGr-SFGl	8.20	0.000	IFGoperr-toMTGr	6.01	0.001
pSMGl-AGl	8.13	0.000	aMTGl-toMTGl	6.00	0.000
SFGl-IFGoperl	8.07	0.000	pITGl-TPl	5.87	0.000
pMTGr-AGr	8.01	0.000	aPaHCr-aTFusCr	5.77	0.000
TOFusCr-toITGr	7.90	0.000	pMTGr-TPr	5.76	0.000
IFGtrir-IFGoperl	7.87	0.000	aMTGl-pMTGl	5.76	0.000
PC-SFGr	7.86	0.000	aMTGl-pITGl	5.75	0.000
toMTGr-AGr	7.85	0.000	TPr-aTFusCr	5.73	0.000
aSTGl-aSTGr	7.78	0.000	aITGl-pITGl	5.72	0.000
pPaHCr-aMTGl	7.76	0.000	pMTGr-pTFusCr	5.71	0.000
pMTGl-MedFC	7.67	0.000	aMTGl-toITGl	5.60	0.000
pTFusCl-TOFusCr	7.66	0.000	Precuneous-AGr	5.58	0.001
PC-AGr	7.65	0.000	IFGtrir-MidFGr	5.48	0.002
AGl-pMTGl	7.64	0.000	MidFGr-PC	5.42	0.001
MidFGr-SFGr	7.61	0.000	aITGl-aMTGl	5.42	0.001
TPl-FOrbl	7.59	0.000	SFGl-toMTGr	5.41	0.001
MedFC-pMTGr	7.56	0.000	PC-SFGr	5.39	0.001
pTFusCl-aPaHCl	7.54	0.000	aITGl-pMTGl	5.33	0.001
AGl-pMTGr	7.52	0.000	aMTGr-pITGr	5.31	0.001
SFGl-MedFC	7.47	0.000	pMTGl-TPl	5.24	0.001
FOrbl-IFGtrir	7.45	0.000	aMTGl-pTFusCl	5.24	0.001
SFGl-pMTGl	7.43	0.000	pMTGl-pTFusCl	5.21	0.001
aMTGr-AGl	7.37	0.000	aITGr-pMTGr	5.20	0.001
pTFusCr-aPaHCl	7.36	0.000	aPaHCl-pPaHCr	5.18	0.001
AGr-MidFGr	7.34	0.000	toITGr-MidFGl	5.14	0.001
pSTGl-aSTGr	7.29	0.000	pMTGl-toITGl	5.12	0.001
IFGoperl-pMTGl	7.28	0.000	aTFusCl-TPl	5.04	0.003
IFGtril-IFGoperl	7.17	0.000	aSMGl-pSMGl	5.03	0.006
pTFusCr-pITGr	7.15	0.000	IFGoperr-pSMGr	4.95	0.003
TPl-aPaHCl	7.15	0.000	pMTGr-aSTGr	4.93	0.001
pSTGl-TPl	7.12	0.000	MidFGr-toMTGl	4.78	0.002
pSMGl-IFGtrir	7.12	0.000	aITGr-pTFusCr	4.78	0.002
aITGl-aITGr	7.12	0.000	TPl-toITGl	4.76	0.002
pSTGr-aSTGl	7.09	0.000	IFGoperr-aSMGr	4.70	0.004
toITGr-toITGl	7.07	0.000	aPaHCl-TPl	4.70	0.002
PC-aMTGl	7.07	0.000	pITGr-FOrbl	4.63	0.001
TOFusCl-toITGr	7.06	0.000	aITGr-toMTGr	4.63	0.003
SFGr-aMTGr	7.02	0.000	MidFGr-Precuneous	4.61	0.003
aITGr-aPaHCr	7.00	0.000	aITGl-TPl	4.58	0.002
pTFusCr-aPaHCr	7.00	0.000	pTFusCl-aTFusCr	4.49	0.002
aTFusCl-aTFusCr	6.98	0.000	MidFGr-AGr	4.37	0.004
aMTGl-AGl	6.93	0.000	pITGr-TPr	4.33	0.002
pSTGr-aSTGr	6.93	0.000	MidFGr-toITGl	4.32	0.004
aSMGl-pSMGr	6.92	0.000	TPr-aSTGr	4.32	0.003

IFGtril-toMTGl	6.91	0.000	MidFGr-FOrbr	4.26	0.004
TPl-pMTGl	6.90	0.000	pTFusCr-MidFGl	4.25	0.003
aMTGl-pITGl	6.89	0.000	IFGtrir-SFGr	4.25	0.014
pPaHCr-Precuneous	6.88	0.000	toMTGl-TPl	4.22	0.003
aTFusCl-aPaHCr	6.88	0.000	aTFusCl-aTFusCr	4.14	0.012
TPl-aMTGr	6.87	0.000	IFGoperr-SFGr	4.11	0.009
SFGl-PC	6.83	0.000	TPl-aTFusCr	4.11	0.004
aITGr-pTFusCr	6.83	0.000	PC-toMTGr	4.10	0.008
IFGoperl-FOrbl	6.83	0.000	IFGoperr-toITGr	4.01	0.009
aTFusCr-pTFusCl	6.81	0.000	toITGl-IFGtril	3.99	0.005
IFGoperr-aSMGr	6.80	0.000	aPaHCl-TPr	3.90	0.010
aSTGr-TOFusCr	6.72	0.000	toITGl-TOFusCl	3.88	0.006
pSTGl-pMTGl	6.70	0.000	PC-SFGl	3.88	0.010
pITGr-pITGl	6.68	0.000	IFGoperr-MidFGr	3.84	0.011
pMTGl-pITGl	6.68	0.000	MidFGr-pSMGr	3.80	0.007
IFGtril-MidFGl	6.64	0.000	aPaHCl-pTFusCl	3.80	0.010
aTFusCr-aPaHCr	6.63	0.000	pMTGl-IFGoperl	3.77	0.009
aSTGl-TPl	6.63	0.000	SFGl-toITGr	3.76	0.016
IFGoperr-IFGtrir	6.61	0.000	aITGr-TPr	3.75	0.013
pMTGr-pITGr	6.60	0.000	TPr-pSTGr	3.72	0.008
IFGtrir-SFGr	6.60	0.000	toITGr-TOFusCr	3.71	0.008
SFGl-pMTGr	6.57	0.000	IFGoperr-aSTGr	3.70	0.013
aMTGl-pMTGr	6.56	0.000	aITGr-MidFGl	3.70	0.013
AGl-MidFGl	6.55	0.000	TPr-toITGr	3.68	0.008
pTFusCr-TOFusCl	6.49	0.000	toITGr-TPr	3.68	0.008
FOrbl-IFGtril	6.49	0.000	Precuneous-toMTGr	3.67	0.024
aITGr-pTFusCl	6.46	0.000	toMTGr-MidFGl	3.66	0.008
pTFusCl-TPl	6.33	0.000	TPl-aSTGl	3.66	0.009
pMTGr-pSTGr	6.32	0.000	aPaHCr-aTFusCl	3.65	0.012
aSMGl-IFGoperr	6.29	0.000	aMTGr-toITGr	3.65	0.020
aSTGl-pSTGl	6.29	0.000	pMTGr-pSTGr	3.61	0.010
aSTGl-pTFusCl	6.27	0.000	IFGtrir-PC	3.58	0.038
IFGtril-pMTGl	6.26	0.000	toITGr-pSMGr	3.55	0.009
aPaHCl-aTFusCr	6.25	0.000	pTFusCr-pPaHCr	3.54	0.010
aSMGl-aSMGr	6.24	0.000	Precuneous-SFGr	3.53	0.025
FOrbr-SFGr	6.24	0.000	aTFusCl-TPr	3.50	0.027
aITGl-aTFusCl	6.22	0.000	pMTGr-pPaHCr	3.45	0.011
FOrbl-MidFGl	6.22	0.000	MidFGr-pTFusCl	3.45	0.014
aSTGl-FOrbr	6.21	0.000	PC-pPaHCr	3.40	0.021
MidFGr-SFGl	6.19	0.000	pMTGr-MidFGl	3.40	0.011
aPaHCl-TPr	6.15	0.000	IFGtrir-AGr	3.38	0.038
pPaHCl-pTFusCl	6.14	0.000	toMTGl-IFGtril	3.36	0.015
FOrbl-pMTGl	6.14	0.000	aITGr-aMTGr	3.36	0.021
pMTGl-pSTGr	6.11	0.000	aITGr-aTFusCr	3.35	0.021
pITGl-pTFusCl	6.11	0.000	PC-pPaHCl	3.34	0.021

IFGoperr-TOFusCl	6.11	0.000	pTFusCr-TPr	3.33	0.014
IFGoperl-pSTGl	6.11	0.000	aPaHCl-aTFusCr	3.33	0.024
aITGr-aPaHCl	6.11	0.000	toMTGl-IFGoperl	3.32	0.015
aMTGr-pMTGl	6.11	0.000	aITGl-MidFGr	3.31	0.018
aSMGr-TOFusCr	6.11	0.000	Precuneous-IFGtril	3.27	0.037
PC-pMTGl	6.10	0.000	aITGr-IFGtrir	3.26	0.023
aMTGr-TPr	6.08	0.000	IFGtrir-FOrbr	3.25	0.038
pMTGr-Precuneous	6.06	0.000	aITGl-IFGtril	3.24	0.018
pITGl-pMTGr	6.04	0.000	toMTGr-TOFusCr	3.23	0.017
IFGtrir-toMTGr	6.04	0.000	aMTGr-MedFC	3.22	0.030
AGr-aMTGr	6.03	0.000	TPl-FOrbr	3.20	0.018
AGl-FOrbl	6.00	0.000	pMTGl-pSTGl	3.17	0.025
AGl-MedFC	6.00	0.000	pMTGr-FOrbl	3.15	0.018
MidFGl-pMTGl	5.98	0.000	pITGl-IFGoperl	3.15	0.022
IFGtril-IFGtrir	5.98	0.000	aITGr-aPaHCl	3.15	0.025
AGl-AGr	5.97	0.000	IFGoperr-SFGl	3.13	0.037
aSMGr-toITGr	5.97	0.000	pTFusCl-pPaHCl	3.12	0.017
aTFusCr-pTFusCr	5.95	0.000	toITGr-aSMGr	3.12	0.022
IFGtril-pSMGl	5.94	0.000	aPaHCr-TPl	3.12	0.030
aITGr-aTFusCl	5.92	0.000	aITGr-aPaHCr	3.12	0.025
pTFusCr-toITGr	5.88	0.000	toITGl-IFGoperl	3.11	0.026
pPaHCl-pTFusCr	5.88	0.000	pMTGl-IFGtril	3.09	0.027
pMTGl-toMTGl	5.87	0.000	aMTGr-MidFGl	3.08	0.030
aSMGr-TOFusCl	5.85	0.000	pTFusCl-TOFusCl	3.06	0.018
IFGoperl-pSMGl	5.83	0.000	aMTGr-pTFusCr	3.06	0.030
aTFusCl-pTFusCr	5.81	0.000	TPl-FOrbl	3.05	0.021
aPaHCr-TPl	5.79	0.000	pITGl-FOrbr	3.05	0.025
aMTGr-Precuneous	5.76	0.000	aMTGr-aSTGr	3.04	0.030
aITGl-pITGl	5.74	0.001	pTFusCr-FOrbl	3.03	0.020
FOrbr-MidFGr	5.74	0.000	toMTGr-FOrbl	3.02	0.025
IFGtril-SFGl	5.74	0.000	toMTGr-IFGtrir	2.97	0.026
aITGr-aTFusCr	5.72	0.000	IFGoperr-IFGtrir	2.96	0.047
AGl-pITGl	5.71	0.000	pMTGr-SFGr	2.91	0.028
pTFusCl-toITGl	5.70	0.000	IFGoperr-pITGr	2.91	0.047
IFGoperr-toMTGl	5.69	0.000	toITGr-IFGtrir	2.88	0.031
SFGl-TPl	5.67	0.000	pTFusCl-IFGtril	2.88	0.023
pMTGr-TPl	5.65	0.000	TPl-pPaHCl	2.87	0.028
AGl-SFGr	5.65	0.000	pITGr-SFGl	2.87	0.028
pPaHCr-TPl	5.64	0.000	PC-MidFGl	2.87	0.049
FOrbr-pMTGr	5.64	0.000	aMTGr-FOrbl	2.87	0.037
pSTGr-TOFusCr	5.62	0.000	pITGl-IFGtril	2.86	0.035
aPaHCl-pPaHCl	5.62	0.000	TPr-pPaHCr	2.85	0.032
FOrbr-IFGoperr	5.62	0.000	PC-TOFusCl	2.84	0.049
pSMGr-toITGr	5.61	0.000	toMTGr-aSTGr	2.83	0.033
pSMGl-pSMGr	5.61	0.000	pITGr-pPaHCr	2.80	0.030

IFGoperr-pSMGl	5.60	0.000
pTFusCr-TPl	5.59	0.000
AGr-pITGr	5.59	0.000
FOrbl-pMTGr	5.55	0.000
IFGoperr-toITGr	5.53	0.000
TOFusCl-toITGl	5.51	0.000
pTFusCl-toMTGr	5.50	0.000
aMTGr-pPaHCl	5.50	0.000
FOrbl-SFGr	5.50	0.000
TOFusCr-toITGl	5.48	0.000
IFGtrir-SFGl	5.48	0.000
IFGtrir-MidFGr	5.44	0.000
IFGtrir-toMTGl	5.44	0.000
pTFusCl-toMTGl	5.43	0.000
aSTGr-TPr	5.42	0.001
Precuneous-SFGr	5.41	0.000
IFGtrir-TPr	5.39	0.000
aSTGl-TOFusCr	5.38	0.000
aMTGl-TPr	5.37	0.000
FOrbr-IFGoperl	5.37	0.000
pMTGl-SFGr	5.36	0.000
aITGl-aPaHCl	5.36	0.001
pTFusCr-toITGl	5.35	0.000
MidFGl-SFGr	5.33	0.000
TOFusCl-TPr	5.31	0.000
MidFGl-pSMGl	5.31	0.000
pTFusCr-TOFusCr	5.28	0.000
IFGoperl-IFGoperr	5.28	0.000
pSMGl-pSTGl	5.27	0.000
IFGtrir-TOFusCl	5.27	0.000
IFGoperl-TPl	5.24	0.000
AGr-pMTGl	5.23	0.000
aSMGr-toITGl	5.23	0.001
IFGoperl-toITGl	5.21	0.000
MidFGr-pMTGr	5.20	0.001
IFGoperl-toMTGr	5.20	0.000
IFGoperr-TOFusCr	5.17	0.000
pPaHCr-pTFusCr	5.16	0.001
AGr-Precuneous	5.16	0.000
aSTGr-TPl	5.16	0.001
pSTGl-TPr	5.15	0.001
pPaHCl-SFGl	5.15	0.001
pSMGl-SFGl	5.13	0.001
pSMGl-toMTGl	5.12	0.001
aSMGl-toITGl	5.12	0.001
aITGr-FOrbl	2.80	0.042
toITGr-Precuneous	2.78	0.035
pMTGr-TOFusCr	2.77	0.033
pTFusCr-aPaHCr	2.77	0.032
toITGl-TOFusCr	2.76	0.043
toITGr-PC	2.73	0.036
MidFGr-aSMGr	2.73	0.046
MidFGr-SFGl	2.69	0.046
pMTGr-MedFC	2.69	0.037
aITGr-aSTGr	2.68	0.048
pTFusCl-aTFusCl	2.67	0.032
toMTGr-aMTGr	2.59	0.049
toITGr-aSTGr	2.58	0.047
toMTGr-SFGr	2.57	0.049
pTFusCr-aSTGr	2.56	0.044
toMTGr-pSMGr	2.55	0.049
pMTGr-SFGl	2.54	0.048
pTFusCr-IFGoperr	2.54	0.044
toITGr-AGr	2.54	0.048
pITGr-aSMGl	2.52	0.049
pTFusCr-aSMGl	2.47	0.048
pTFusCr-aPaHCl	2.45	0.048
Anticorrelations		
toMTGl-pTFusCr	-5.94	0.000
pTFusCl-pITGr	-5.55	0.000
pITGl-pTFusCr	-5.40	0.000
pTFusCr-pMTGl	-4.96	0.001
pITGr-aMTGl	-4.86	0.001
pTFusCl-pTFusCr	-4.77	0.001
pITGr-toMTGl	-4.66	0.001
pMTGr-pTFusCl	-4.50	0.002
aPaHCr-aSMGl	-4.42	0.003
toMTGr-pTFusCl	-4.38	0.002
pTFusCr-aMTGl	-4.34	0.002
pTFusCl-MidFGl	-4.00	0.004
aMTGl-aMTGr	-3.87	0.006
pITGl-pITGr	-3.86	0.007
pTFusCl-aSMGl	-3.80	0.005
aPaHCr-IFGoperl	-3.76	0.011
pTFusCr-aITGl	-3.72	0.007
toITGr-pTFusCl	-3.70	0.008
pMTGr-aITGl	-3.62	0.010
pITGl-toMTGr	-3.50	0.014
aITGl-aSMGl	-3.46	0.015
pITGl-pMTGr	-3.45	0.014

toITGl-toMTGl	5.11	0.001
aITGr-pITGl	5.10	0.001
aMTGr-pITGr	5.10	0.000
IFGtrir-pSMGr	5.09	0.000
IFGtril-TPr	5.08	0.001
aTFusCl-TPl	5.06	0.001
toMTGl-TPl	5.05	0.001
pSMGl-toMTGr	5.04	0.001
AGl-PC	5.04	0.000
aITGl-pTFusCl	5.03	0.001
pITGl-pTFusCr	5.02	0.001
pTFusCl-TPr	5.01	0.000
MidFGr-pSMGl	4.99	0.001
AGr-pSMGl	4.99	0.001
aMTGl-pITGr	4.98	0.001
pITGl-TPl	4.97	0.001
pSTGr-TPl	4.95	0.001
pMTGr-pSMGl	4.95	0.000
MedFC-SFGr	4.95	0.001
pSTGl-TOFusCl	4.91	0.001
aSTGr-FOrbr	4.91	0.001
FOrbl-toMTGr	4.91	0.001
AGr-SFGl	4.90	0.001
aTFusCr-TPr	4.90	0.001
aITGl-aMTGl	4.89	0.001
pITGr-SFGr	4.88	0.001
MedFC-pPaHCl	4.88	0.001
pSMGr-TOFusCl	4.86	0.001
Precuneous-pTFusCl	4.86	0.001
SFGr-toMTGr	4.85	0.001
PC-pPaHCr	4.85	0.001
IFGtrir-MidFGl	4.85	0.001
pSTGr-toMTGl	4.84	0.001
pPaHCr-pTFusCl	4.83	0.001
aMTGl-pPaHCl	4.82	0.001
PC-TPl	4.80	0.001
IFGtrir-TPl	4.80	0.001
TOFusCr-toMTGr	4.79	0.001
TOFusCl-toMTGr	4.79	0.001
aPaHCr-pPaHCl	4.78	0.001
IFGtril-toMTGr	4.77	0.001
pMTGr-toMTGr	4.76	0.001
aPaHCr-pTFusCl	4.75	0.001
aSTGl-TOFusCl	4.75	0.001
aPaHCr-pPaHCr	4.74	0.001

pMTGr-toMTGl	-3.41	0.011
pMTGl-pITGr	-3.41	0.017
TPl-aSMGl	-3.38	0.015
TPl-pITGr	-3.32	0.015
pITGl-aSMGl	-3.31	0.017
aMTGr-aITGl	-3.27	0.030
aSMGl-FOrbr	-3.20	0.036
pTFusCr-toITGl	-3.16	0.019
aSMGl-aMTGl	-3.15	0.036
toMTGl-aMTGr	-3.14	0.021
TPl-pTFusCr	-3.06	0.021
pITGr-aITGl	-3.03	0.023
aPaHCr-pSMGl	-2.98	0.037
aSMGl-aPaHCl	-2.98	0.044
pTFusCl-aMTGr	-2.95	0.022
TPr-IFGoperl	-2.95	0.027
toMTGl-TPr	-2.95	0.029
aMTGl-toITGr	-2.95	0.036
toITGl-aSMGl	-2.94	0.034
aITGr-pTFusCl	-2.91	0.037
pTFusCl-SFGl	-2.86	0.023
pMTGr-aMTGl	-2.84	0.030
toITGr-TPl	-2.83	0.033
pITGr-toITGl	-2.82	0.030
MidFGr-pTFusCr	-2.77	0.045
MidFGr-aSMGl	-2.76	0.045
toMTGl-aITGr	-2.68	0.048
aITGr-aMTGl	-2.63	0.049
pTFusCr-FOrbr	-2.60	0.043
TPl-MidFGl	-2.59	0.046

aSTGr-pMTGl	4.74	0.001
aPaHCl-pPaHCr	4.73	0.001
aTFusCl-pTFusCl	4.73	0.001
pMTGr-pSTGl	4.72	0.001
MidFGl-PC	4.72	0.001
MidFGr-toMTGr	4.70	0.001
aSTGr-pPaHCr	4.70	0.001
SFGl-toMTGl	4.69	0.001
SFGl-toMTGr	4.67	0.001
aMTGr-MidFGl	4.67	0.001
FOrbl-TOFusCr	4.67	0.001
IFGtril-toITGl	4.66	0.001
AGl-TPl	4.64	0.001
aMTGr-pITGl	4.64	0.001
FOrbr-TPl	4.64	0.001
aMTGl-pSTGr	4.63	0.001
FOrbr-pSMGr	4.63	0.001
TOFusCl-TPl	4.62	0.001
pITGr-TOFusCl	4.61	0.001
pITGl-SFGl	4.61	0.001
MidFGr-toMTGl	4.61	0.001
aMTGl-aPaHCl	4.61	0.001
pPaHCl-TOFusCl	4.60	0.001
pITGr-toMTGr	4.60	0.001
aITGl-TPl	4.58	0.002
aITGl-pITGr	4.57	0.002
aSTGr-FOrbl	4.57	0.002
FOrbr-pSMGl	4.57	0.001
pPaHCl-TPl	4.55	0.001
FOrbl-pSMGl	4.54	0.001
pSMGl-pSTGr	4.52	0.001
pMTGr-TPr	4.51	0.001
FOrbl-TOFusCl	4.50	0.001
FOrbl-IFGoperr	4.49	0.001
aITGl-aTFusCr	4.48	0.002
aMTGl-aSTGl	4.48	0.001
pSTGl-TOFusCr	4.47	0.002
IFGoperl-TPr	4.47	0.001
aITGr-TPl	4.43	0.002
IFGtril-pSTGl	4.43	0.002
aMTGr-toMTGr	4.42	0.001
pMTGl-pSMGl	4.41	0.001
AGr-IFGtrir	4.41	0.002
aSTGr-IFGoperl	4.41	0.002
AGr-FOrbr	4.40	0.002

IFGtril-TPl	4.39	0.002
pSTGl-toMTGl	4.38	0.002
MidFGl-toMTGl	4.36	0.002
pITGl-toMTGr	4.35	0.002
aITGl-pMTGr	4.35	0.002
pMTGr-pPaHCr	4.34	0.001
aMTGl-FOrbl	4.33	0.001
AGl-pITGr	4.32	0.002
FOrbl-pSTGl	4.32	0.001
IFGtril-MidFGr	4.32	0.002
aMTGr-MidFGr	4.31	0.001
pSTGr-toMTGr	4.28	0.002
Precuneous-pTFusCr	4.28	0.003
MidFGr-Precuneous	4.27	0.002
aITGr-aMTGl	4.27	0.002
aITGr-FOrbr	4.27	0.002
pITGr-pTFusCl	4.25	0.002
aSTGl-toMTGr	4.25	0.003
MidFGr-PC	4.24	0.002
aMTGr-FOrbl	4.24	0.001
aMTGr-FOrbr	4.24	0.001
toITGl-toMTGr	4.20	0.003
pSMGl-SFGr	4.20	0.002
aSTGr-TOFusCl	4.20	0.003
SFGr-toMTGl	4.18	0.002
MidFGr-toITGl	4.18	0.002
IFGoperr-SFGr	4.18	0.002
aITGl-pTFusCr	4.17	0.003
FOrbl-MedFC	4.17	0.002
PC-pITGl	4.16	0.002
aSMGr-FOrbr	4.16	0.004
toITGr-toMTGr	4.15	0.004
pSMGr-toMTGl	4.15	0.003
IFGtrir-pTFusCl	4.15	0.002
IFGoperr-MidFGr	4.15	0.002
pTFusCl-toITGr	4.14	0.002
PC-pITGr	4.14	0.002
aSTGl-TPr	4.13	0.003
aSTGl-pMTGl	4.12	0.003
aSTGl-toMTGl	4.12	0.003
FOrbl-pSTGr	4.12	0.002
aSMGl-pSMGl	4.11	0.006
pSMGr-toITGl	4.10	0.003
pPaHCr-TOFusCl	4.09	0.003
MidFGr-pSMGr	4.08	0.002

pSMGr-toMTGr	4.07	0.003
AGr-pSMGr	4.07	0.003
aMTGr-pPaHCr	4.06	0.002
pMTGl-TPr	4.05	0.002
MidFGl-TPl	4.03	0.003
aMTGr-IFGtrir	4.01	0.002
pMTGr-toMTGl	4.00	0.002
IFGoperl-MidFGr	4.00	0.003
AGr-toMTGl	4.00	0.003
FOrbl-PC	4.00	0.002
pTFusCr-TPr	3.99	0.003
AGl-IFGtril	3.99	0.003
aSTGr-toMTGl	3.99	0.004
FOrbr-pMTGl	3.99	0.003
Precuneous-toMTGr	3.98	0.004
aSMGl-FOrbr	3.98	0.007
MidFGl-pMTGr	3.97	0.003
AGl-toMTGl	3.97	0.003
AGr-MidFGl	3.97	0.003
aSMGr-IFGoperl	3.97	0.005
aTFusCl-pPaHCr	3.97	0.006
FOrbr-toMTGr	3.97	0.003
pSTGl-toMTGr	3.94	0.004
IFGoperr-toITGl	3.94	0.003
FOrbl-pITGl	3.94	0.002
pITGr-toITGr	3.93	0.004
aMTGl-Precuneous	3.93	0.003
IFGoperr-TPr	3.92	0.003
aSTGr-pMTGr	3.92	0.004
pTFusCr-toMTGr	3.91	0.003
MidFGl-Precuneous	3.91	0.003
FOrbr-pSTGr	3.91	0.003
MidFGr-pITGr	3.90	0.003
IFGtrir-pMTGr	3.90	0.003
aMTGr-IFGtril	3.90	0.003
FOrbr-TOFusCl	3.89	0.003
aSTGr-pPaHCl	3.87	0.004
aSTGl-toITGl	3.86	0.005
pTFusCr-toMTGl	3.84	0.003
pPaHCr-TOFusCr	3.84	0.004
MidFGr-pMTGl	3.83	0.003
AGl-toMTGr	3.82	0.004
aITGr-toMTGr	3.82	0.005
aPaHCr-FOrbr	3.82	0.006
TOFusCl-toMTGl	3.80	0.003

AGr-MedFC	3.80	0.004
aSMGl-IFGoperl	3.80	0.009
pITGr-pMTGl	3.79	0.004
aMTGr-aPaHCr	3.79	0.003
MedFC-Precuneous	3.78	0.005
aMTGl-pTFusCl	3.78	0.004
aSTGr-IFGtrir	3.77	0.005
pITGr-toITGl	3.76	0.004
IFGoperl-SFGr	3.76	0.004
AGr-pITGl	3.76	0.004
FOrbr-pITGr	3.76	0.003
IFGtrir-pSTGl	3.75	0.004
aMTGr-pTFusCl	3.75	0.003
Precuneous-SFGl	3.74	0.006
MidFGl-toITGl	3.74	0.005
aPaHCl-TOFusCl	3.73	0.007
toMTGr-TPl	3.72	0.003
pPaHCl-TOFusCr	3.72	0.006
aTFusCl-pITGl	3.72	0.009
FOrbr-PC	3.71	0.004
pSMGr-TOFusCr	3.70	0.006
IFGtrir-pSTGr	3.70	0.004
pSMGl-toITGl	3.69	0.004
AGl-IFGtrir	3.69	0.005
IFGtril-pTFusCl	3.68	0.006
pSMGr-SFGr	3.66	0.006
aSMGl-IFGtrir	3.66	0.011
FOrbr-TOFusCr	3.66	0.004
pSTGr-TOFusCl	3.65	0.006
AGl-TPr	3.65	0.005
pSMGl-TPl	3.64	0.005
pITGl-toMTGl	3.64	0.006
MidFGr-toITGr	3.64	0.005
pMTGl-toMTGr	3.63	0.004
AGl-MidFGr	3.63	0.005
MedFC-TPl	3.62	0.007
IFGoperl-pSMGr	3.62	0.005
pPaHCl-TPr	3.61	0.007
aMTGr-pSTGl	3.61	0.004
aSTGr-IFGoperr	3.61	0.006
aTFusCr-pITGl	3.61	0.012
aMTGl-pSTGl	3.60	0.005
aMTGl-SFGr	3.60	0.005
pITGr-TOFusCr	3.59	0.006
aMTGl-MidFGl	3.59	0.005

aSMGr-aSTGl	3.59	0.010
aSTGl-aTFusCl	3.59	0.007
aPaHCl-pSTGl	3.58	0.008
MedFC-pITGl	3.57	0.007
aITGl-pMTGl	3.57	0.010
FOrbr-SFGl	3.57	0.004
IFGtril-pSTGr	3.57	0.007
pSMGl-TPr	3.56	0.005
IFGoperr-pTFusCl	3.56	0.006
aMTGl-toMTGr	3.56	0.005
FOrbl-pSMGr	3.56	0.005
toITGr-toMTGl	3.54	0.010
IFGtril-SFGr	3.54	0.007
PC-toMTGr	3.53	0.006
aMTGr-pSTGr	3.53	0.005
aSTGl-pTFusCr	3.53	0.008
aSTGr-PC	3.53	0.007
aTFusCl-TPr	3.53	0.011
TOFusCr-toMTGl	3.52	0.007
IFGtrir-pMTGl	3.52	0.005
aITGl-toMTGl	3.52	0.010
aMTGr-aPaHCl	3.52	0.005
aPaHCl-pITGl	3.52	0.008
aPaHCr-pITGr	3.52	0.010
AGl-IFGoperl	3.50	0.006
aMTGr-aSTGr	3.50	0.005
Precuneous-TPl	3.49	0.008
pSTGr-AGl	3.49	0.007
AGl-pSTGr	3.49	0.006
aSMGr-IFGtrir	3.49	0.012
aSTGr-Precuneous	3.49	0.007
IFGtril-TOFusCl	3.49	0.007
IFGoperl-pSTGr	3.47	0.006
aSMGl-toITGr	3.47	0.015
TOFusCr-TPr	3.46	0.007
FOrbl-toMTGl	3.46	0.006
pITGl-SFGr	3.45	0.007
aSTGr-toMTGr	3.45	0.007
pMTGr-pPaHCl	3.43	0.006
IFGoperl-TOFusCr	3.43	0.006
IFGoperl-TOFusCl	3.42	0.006
MedFC-MidFGl	3.41	0.009
aPaHCl-pITGr	3.40	0.010
pSMGr-pSTGr	3.39	0.009
FOrbr-IFGtril	3.39	0.006

IFGtril-pMTGr	3.37	0.008
toMTGr-TPr	3.36	0.005
pITGl-pSMGl	3.36	0.008
aSTGl-FOrbl	3.35	0.011
aTFusCr-TPl	3.35	0.019
MidFGl-pITGl	3.34	0.009
AGl-Precuneous	3.33	0.008
SFGr-TPl	3.31	0.008
MidFGr-pITGl	3.31	0.009
aSMGr-pTFusCr	3.31	0.016
pSTGl-SFGl	3.30	0.011
aITGl-aPaHCr	3.30	0.015
aPaHCr-aSTGr	3.30	0.013
aPaHCr-FOrbl	3.30	0.013
pSMGl-TOFusCr	3.27	0.009
pITGl-toITGl	3.27	0.009
MidFGl-pSTGl	3.26	0.010
aITGr-pMTGr	3.26	0.016
FOrbl-toITGl	3.26	0.008
PC-pSTGr	3.25	0.011
IFGoperr-IFGtril	3.25	0.012
IFGtrir-TOFusCr	3.24	0.008
Precuneous-pSTGr	3.23	0.012
AGr-FOrbl	3.23	0.012
aSTGl-IFGoperl	3.23	0.013
aPaHCl-Precuneous	3.22	0.013
PC-pTFusCl	3.20	0.011
aPaHCl-FOrbl	3.20	0.013
pMTGl-Precuneous	3.19	0.009
aSMGl-TOFusCl	3.19	0.024
IFGtrir-toITGl	3.18	0.009
TOFusCr-TPl	3.17	0.011
pSMGr-pTFusCr	3.17	0.014
aSTGl-pPaHCr	3.17	0.014
IFGoperl-toITGr	3.16	0.010
aMTGl-TOFusCl	3.16	0.011
MedFC-pPaHCr	3.15	0.015
SFGl-TPr	3.13	0.012
pSTGl-SFGr	3.13	0.013
IFGoperl-pMTGr	3.13	0.011
aITGl-SFGl	3.13	0.020
aSMGr-FOrbl	3.12	0.022
aMTGl-pTFusCr	3.11	0.011
FOrbr-pSTGl	3.11	0.011
pSTGl-pTFusCr	3.10	0.013

pITGr-pSMGl	3.10	0.014
aPaHCl-PC	3.10	0.016
IFGoperr-pTFusCr	3.08	0.016
aSTGr-pTFusCr	3.08	0.015
FOrbl-pITGr	3.08	0.010
FOrbl-pTFusCr	3.08	0.010
FOrbr-MidFGl	3.08	0.011
Precuneous-TOFusCl	3.06	0.016
pPaHCr-pSTGl	3.06	0.019
aPaHCr-pSTGr	3.06	0.020
SFGr-TPr	3.05	0.013
pITGr-pSTGl	3.05	0.014
pPaHCr-toITGl	3.04	0.019
aMTGl-aSTGr	3.04	0.013
FOrbr-pITGl	3.04	0.012
pSMGl-toITGr	3.00	0.014
pITGr-toMTGl	3.00	0.014
aPaHCl-toITGl	3.00	0.019
aSMGr-pTFusCl	3.00	0.026
aTFusCl-pITGr	3.00	0.030
IFGtrir-toITGr	2.99	0.013
pITGr-SFGl	2.98	0.014
aSTGl-IFGoperr	2.98	0.019
MidFGr-pSTGl	2.97	0.017
aSTGr-pTFusCl	2.96	0.017
toMTGl-TPr	2.95	0.014
aMTGl-IFGtril	2.95	0.015
FOrbr-toMTGl	2.94	0.014
MidFGl-toMTGr	2.93	0.019
AGr-aMTGl	2.93	0.021
aPaHCl-AGl	2.92	0.021
AGl-aPaHCl	2.92	0.018
aITGl-aMTGr	2.92	0.027
aTFusCr-pITGr	2.92	0.042
pSTGl-AGl	2.91	0.017
AGl-pSTGl	2.91	0.018
aITGl-toMTGr	2.91	0.027
aMTGl-aPaHCr	2.91	0.015
aSMGl-toMTGl	2.91	0.039
FOrbl-MidFGr	2.90	0.013
pPaHCr-TPr	2.89	0.024
IFGoperl-pTFusCl	2.89	0.017
AGl-aITGl	2.89	0.018
aSMGr-toMTGl	2.89	0.031
pSTGr-SFGl	2.88	0.020

MidFGr-pTFusCl	2.88	0.019
pITGl-TOFusCl	2.87	0.018
pSMGl-TOFusCl	2.85	0.019
pITGl-TOFusCr	2.85	0.019
pSTGr-pTFusCl	2.83	0.021
pSTGl-pTFusCl	2.83	0.019
pPaHCr-toMTGr	2.83	0.027
MedFC-pITGr	2.83	0.028
aSTGl-IFGtril	2.83	0.025
pITGr-pSMGr	2.82	0.018
Precuneous-TOFusCr	2.81	0.025
aSMGr-pSMGl	2.81	0.034
aTFusCl-pPaHCl	2.81	0.041
MedFC-TPr	2.79	0.029
AGl-FOrbr	2.79	0.022
FOrbl-pTFusCl	2.79	0.016
aSTGr-IFGtril	2.78	0.023
IFGoperl-pITGl	2.77	0.021
aSTGr-SFGl	2.77	0.023
aMTGl-TOFusCr	2.76	0.020
aSTGl-pMTGr	2.76	0.027
aSTGl-SFGl	2.75	0.027
aSTGr-toITGl	2.75	0.024
pITGr-TPl	2.73	0.022
aSTGl-toITGr	2.72	0.028
toITGl-TPl	2.71	0.028
AGl-pTFusCl	2.71	0.025
aSTGr-pSMGr	2.71	0.024
pMTGr-pTFusCl	2.70	0.022
IFGtrir-pITGl	2.70	0.023
aPaHCl-aSTGr	2.70	0.032
aSMGr-IFGtril	2.70	0.040
aITGr-TPr	2.69	0.050
aMTGl-toMTGl	2.69	0.022
aPaHCr-pMTGr	2.69	0.039
pMTGr-TOFusCl	2.68	0.022
aPaHCr-pSTGl	2.67	0.039
toMTGl-aTFusCl	2.67	0.021
aITGl-aSTGl	2.66	0.041
IFGtrir-PC	2.64	0.026
aPaHCl-aSTGl	2.64	0.034
pTFusCl-SFGl	2.63	0.023
MedFC-MidFGr	2.61	0.038
AGr-TPl	2.61	0.040
Precuneous-pSTGl	2.60	0.037

aSTGl-IFGtrir	2.60	0.032
aSMGr-aSTGr	2.59	0.046
pSMGr-TPr	2.58	0.040
pMTGl-toITGl	2.58	0.031
FOrbl-aITGr	2.58	0.025
Precuneous-toMTGl	2.57	0.038
aMTGr-pSMGl	2.56	0.031
pTFusCl-aSMGl	2.56	0.025
aSTGl-pPaHCl	2.56	0.033
FOrbl-aSMGl	2.55	0.025
FOrbr-toITGr	2.55	0.029
aPaHCr-pITGl	2.54	0.047
aSMGr-MidFGr	2.54	0.046
FOrbr-pTFusCl	2.54	0.029
aMTGr-aSTGl	2.53	0.032
aSMGr-TPr	2.53	0.046
FOrbr-MedFC	2.53	0.029
aSMGr-toMTGr	2.52	0.046
pMTGl-TOFusCr	2.51	0.035
IFGtrir-pPaHCr	2.51	0.032
toITGl-aTFusCl	2.51	0.039
MidFGr-pTFusCr	2.50	0.038
MidFGl-pSTGr	2.50	0.042
MidFGl-TPr	2.50	0.042
IFGoperr-pITGl	2.50	0.046
aSTGl-pSMGr	2.50	0.035
IFGtril-toITGr	2.50	0.039
AGl-pTFusCr	2.49	0.037
pSMGr-pTFusCl	2.48	0.045
FOrbr-pPaHCr	2.48	0.031
pSTGl-toITGl	2.47	0.036
toITGl-pPaHCl	2.47	0.040
pITGr-Precuneous	2.47	0.035
IFGtril-TOFusCr	2.47	0.040
pITGr-pPaHCl	2.45	0.036
IFGoperr-AGr	2.45	0.048
aMTGr-toMTGl	2.45	0.035
FOrbr-pTFusCr	2.45	0.031
toITGl-aPaHCr	2.43	0.041
MidFGr-aSMGl	2.43	0.042
Precuneous-toITGl	2.42	0.047
pMTGr-pTFusCr	2.42	0.035
IFGtril-pSMGr	2.42	0.042
PC-pSTGl	2.41	0.049
pSTGl-AGr	2.41	0.038

pSTGr-pTFusCr	2.40	0.048
PC-aITGl	2.40	0.049
MidFGr-pPaHCr	2.37	0.046
aSTGl-Precuneous	2.37	0.044
TOFusCr-aSMGl	2.35	0.049
aMTGl-IFGoperl	2.31	0.046
pTFusCr-SFGr	2.29	0.048
IFGtrir-pITGr	2.29	0.047
pITGr-IFGoperr	2.28	0.047
pTFusCl-pSMGl	2.27	0.042
aMTGl-IFGtrir	2.27	0.047
toMTGr-aTFusCl	2.25	0.045
toMTGr-pPaHCl	2.24	0.045
Anticorrelations		
aSMGl-MedFC	-4.46	0.004
aSMGr-MedFC	-4.43	0.002
MedFC-pSMGr	-4.24	0.002
aMTGl-aSMGl	-2.83	0.017
IFGoperr-MedFC	-2.75	0.029
aMTGr-aSMGr	-2.52	0.032
aMTGr-aSMGl	-2.27	0.049

Note. See Table 2 for a key to region abbreviations.
