# Supplementary material for: Hypoconnectivity of Resting-State Networks in Persons with Aphasia Compared with Healthy Age-Matched Adults
Source: Front Hum Neurosci. 2017 Feb 28;11:91. doi: 10.3389/fnhum.2017.00091 (PMC5329062; doi:10.3389/fnhum.2017.00091)
Supplement: Supplementary Table 3 — Connection differences between groups in resting state networks. [file Table3.pdf]

Supplementary Table 3

Connection Differences Between Groups in Resting State Networks

NHA>PWA			PWA>NHA
Connection	T(16)	p-FDR	No significant differences
Default Mode Network			
LLP-MPFC	3.99	0.003	
LLP-PCC	2.31	0.046	
LLP-RLP	2.17	0.046	
Dorsal Attention Network			
iLOCr-iLOCl	5.72	0.000	
iLOCl-aSMGl	5.08	0.000	
PreCGl-iLOCl	4.63	0.002	
PreCGl-PreCGr	4.14	0.003	
iLOCr-PreCGr	3.27	0.017	
iLOCl-sLOCr	2.71	0.025	
iLOCl-aSMGr	2.63	0.025	
Executive Control Network			
AGl-PaCiGl	4.87	0.001	
PaCiGr-AGl	3.25	0.025	
PaCiGr-AGr	2.60	0.048	
Salience Network			
ICr-ICl	6.91	0.000	
pSMGr-ICl	5.70	0.000	
ICl-AC	4.55	0.001	
pSMGl-ICr	3.01	0.050	
pSMGl-FPl	2.64	0.050	
pSMGl-FPr	2.48	0.050	
Sensorimotor Network			
PostCGl-PreCGr	5.43	0.000	
PostCGr-PostCGl	4.66	0.001	
PreCGr-PreCGl	4.14	0.001	
PreCGl-PostCGr	3.56	0.004	
Auditory Network			
PTl-PTr	6.88	0.000	
Visual Network			
No significant differences			

Note. See Table 2 for a key to region abbreviations. NHA = Neurologically Healthy Adults; PWA = Persons with Aphasia.
