# Supplementary material for: Hypoconnectivity of Resting-State Networks in Persons with Aphasia Compared with Healthy Age-Matched Adults
Source: Front Hum Neurosci. 2017 Feb 28;11:91. doi: 10.3389/fnhum.2017.00091 (PMC5329062; doi:10.3389/fnhum.2017.00091)
Supplement: Supplementary Table 4 — Connection differences between groups in the semantic network. [file Table4.pdf]

Supplementary Table 4

Connection Differences Between Groups in The Semantic Network

NHA>PWA			PWA>NHA		
Connection	T(16)	p-FDR	Connection	T(16)	p-FDR
aMTGr-aMTGl	9.37	0.000	pITGl-pTFusCl	-8.13	0.000
pTFusCr-pTFusCl	9.27	0.000	toMTGl-aITGl	-6.79	0.000
pMTGr-pMTGl	7.41	0.000	toITGr-pTFusCr	-5.75	0.001
pTFusCr-pITGl	7.35	0.000	pTFusCr-pITGr	-4.94	0.001
pITGr-pITGl	7.18	0.000	toITGr-pITGr	-4.76	0.003
PC-aMTGr	7.14	0.000	toITGr-pMTGr	-4.34	0.005
pTFusCr-toMTGl	7.03	0.000	pPaHCr-aPaHCr	-4.32	0.008
MedFC-aMTGr	7.01	0.000	aITGr-toITGr	-4.05	0.009
pITGr-pTFusCl	6.99	0.000	pITGl-toMTGl	-3.74	0.008
pITGr-aMTGl	6.91	0.000	pTFusCl-toMTGl	-3.54	0.007
pTFusCl-toMTGr	6.85	0.000	pTFusCl-aITGl	-3.48	0.008
toMTGr-toMTGl	6.65	0.000	pITGr-MidFGl	-3.43	0.012
pITGl-pMTGr	6.47	0.000	toITGl-aITGl	-3.35	0.031
aMTGl-SFGl	6.33	0.000	toITGl-aMTGl	-3.17	0.039
pMTGr-aMTGl	6.32	0.000	TPr-aMTGr	-3.12	0.030
pTFusCl-aITGr	6.30	0.000	toITGr-aMTGr	-3.03	0.041
pPaHCr-aMTGl	5.89	0.001	aMTGl-toMTGl	-3.02	0.025
pTFusCr-TPl	5.88	0.000	pTFusCr-pMTGr	-2.95	0.033
toITGl-pTFusCr	5.80	0.001	toITGr-TPr	-2.91	0.047
FOrbr-aSTGl	5.70	0.002	pITGl-toITGl	-2.86	0.032
aITGl-pMTGr	5.54	0.001	pMTGl-pTFusCl	-2.86	0.035
pTFusCr-aITGl	5.51	0.000	pTFusCr-toMTGr	-2.57	0.050
toMTGl-pITGr	5.51	0.001	pTFusCr-MidFGl	-2.53	0.050
aSMGl-toITGl	5.49	0.002			
toITGr-pTFusCl	5.47	0.001			
pITGl-toMTGr	5.45	0.001			
SFGl-TPl	5.35	0.001			
pTFusCr-aMTGl	5.33	0.000			
aMTGr-SFGl	5.28	0.001			
SFGl-AGl	5.26	0.001			
aITGl-pITGr	5.23	0.001			
MedFC-aMTGl	5.23	0.002			
pTFusCl-pMTGr	5.20	0.001			
pMTGr-toMTGl	5.16	0.001			
pITGl-aITGr	5.12	0.001			
TPr-IFGoperl	5.09	0.003			
pSTGr-pSTGl	5.09	0.005			
aITGl-aITGr	5.08	0.001			
IFGtril-TPr	5.07	0.005			
pMTGl-pITGr	5.03	0.002			
FOrbr-aSMGl	4.98	0.003			

pMTGr-TPl	4.97	0.001
SFGl-pMTGl	4.97	0.001
TPl-aMTGr	4.96	0.002
MidFGr-MidFGl	4.90	0.007
aMTGr-pITGl	4.84	0.001
FOrbl-SFGl	4.81	0.009
aMTGl-aITGr	4.72	0.001
pMTGl-MedFC	4.71	0.002
pMTGl-aMTGr	4.65	0.002
aMTGr-pTFusCl	4.64	0.002
SFGr-aMTGr	4.59	0.014
aMTGl-TPr	4.58	0.002
pTFusCl-aSTGl	4.57	0.002
pTFusCl-aSMGl	4.57	0.002
toITGl-pITGr	4.55	0.005
IFGtrir-IFGoperl	4.55	0.009
MidFGl-TPl	4.54	0.006
IFGoperl-FOrbr	4.51	0.006
AGl-pMTGr	4.50	0.008
pSMGl-IFGtrir	4.44	0.010
pSTGr-pSMGl	4.43	0.010
aMTGr-aITGl	4.38	0.002
MedFC-PC	4.38	0.005
toITGr-toITGl	4.34	0.005
MidFGl-FOrbr	4.33	0.006
TPl-pPaHCr	4.31	0.004
TPl-pITGr	4.29	0.004
toMTGl-IFGoperr	4.29	0.004
aMTGr-AGl	4.27	0.003
TPl-toMTGr	4.26	0.004
toMTGl-TPr	4.14	0.005
toMTGr-aMTGl	4.12	0.007
aSTGr-toMTGl	4.11	0.024
pTFusCl-TPr	4.06	0.004
pPaHCr-aSTGl	4.04	0.011
pMTGl-aSTGr	3.99	0.007
PC-aMTGl	3.99	0.014
toMTGl-aMTGr	3.98	0.006
AGr-aMTGr	3.98	0.050
aMTGl-AGl	3.97	0.004
aMTGr-MidFGr	3.94	0.004
pTFusCl-IFGoperr	3.93	0.004
pTFusCl-SFGl	3.88	0.004
PC-pPaHCl	3.87	0.014
AGl-MedFC	3.83	0.012

PC-pMTGr	3.83	0.014
FOrbl-FOrbr	3.83	0.030
pITGl-AGl	3.80	0.008
aSTGl-aSTGr	3.80	0.018
MidFGl-aMTGl	3.79	0.014
SFGr-pMTGr	3.79	0.036
pMTGl-TPr	3.76	0.010
pTFusCl-TOFusCr	3.75	0.005
pTFusCr-MidFGr	3.72	0.008
MidFGl-pITGl	3.72	0.014
pPaHCl-Precuneous	3.72	0.043
SFGl-pITGl	3.71	0.011
toMTGl-toITGr	3.70	0.008
TPr-TPl	3.69	0.013
aSMGl-pSMGr	3.69	0.016
pTFusCl-TOFusCl	3.68	0.006
AGl-pMTGl	3.67	0.014
MidFGl-pMTGl	3.67	0.014
pMTGr-AGr	3.63	0.009
pITGr-FOrbr	3.62	0.010
MidFGr-pSMGl	3.62	0.020
SFGr-AGl	3.61	0.036
MidFGr-pITGr	3.57	0.020
FOrbr-pTFusCr	3.56	0.017
IFGtrir-pSTGl	3.54	0.042
pSTGr-pMTGl	3.49	0.036
IFGoperl-FOrbl	3.48	0.036
pSTGr-aSTGl	3.48	0.036
FOrbl-pTFusCl	3.47	0.030
FOrbl-pITGl	3.46	0.030
toMTGr-toITGl	3.45	0.023
SFGl-FOrbr	3.44	0.017
MidFGr-pMTGr	3.44	0.020
MidFGr-pSTGl	3.43	0.020
toMTGr-aITGl	3.43	0.023
SFGl-MedFC	3.39	0.017
pSTGl-TPr	3.39	0.036
pSTGl-aSTGr	3.37	0.036
toMTGr-pMTGl	3.36	0.023
toMTGl-SFGl	3.35	0.013
TPl-pSMGl	3.35	0.019
FOrbr-aSMGr	3.35	0.021
MidFGl-toMTGl	3.34	0.021
aSTGr-pPaHCl	3.31	0.041
pITGl-IFGoperr	3.29	0.016

pMTGl-FOrbl	3.27	0.017
aSTGl-TPr	3.26	0.038
TPl-aSTGr	3.21	0.023
toMTGl-aSMGl	3.18	0.017
AGl-pSTGr	3.16	0.031
SFGl-pSMGl	3.15	0.022
pSMGl-SFGr	3.15	0.034
pSMGl-IFGoperr	3.15	0.034
SFGl-IFGoperl	3.14	0.022
pSMGl-IFGtril	3.12	0.034
TPl-PC	3.10	0.025
TPl-MedFC	3.09	0.025
TPl-IFGtrir	3.05	0.025
FOrbr-toMTGr	3.04	0.036
toMTGr-IFGoperl	3.01	0.038
FOrbr-IFGtrir	2.98	0.037
aSMGl-IFGtrir	2.98	0.045
TPl-AGl	2.96	0.028
aMTGr-pPaHCl	2.92	0.027
FOrbr-IFGoperr	2.91	0.039
aITGl-aTFusCl	2.91	0.042
aMTGr-Precuneous	2.90	0.027
toMTGl-aSTGl	2.89	0.027
pTFusCl-aSTGr	2.85	0.025
aMTGl-aSTGr	2.85	0.033
SFGl-aITGl	2.85	0.035
TPr-TOFusCl	2.85	0.045
toMTGl-IFGoperl	2.84	0.029
pTFusCl-pPaHCr	2.83	0.025
pTFusCl-Precuneous	2.82	0.025
pTFusCr-pPaHCl	2.75	0.047
SFGl-IFGtril	2.71	0.045
toMTGl-pSTGl	2.68	0.038
pTFusCr-aPaHCl	2.68	0.050
toMTGl-pSMGl	2.66	0.038
pTFusCr-aSTGl	2.64	0.050
pTFusCr-pSTGl	2.62	0.050
pTFusCl-pSTGl	2.59	0.037
pTFusCl-pSMGl	2.58	0.037
pTFusCr-pSMGr	2.57	0.050
toMTGl-pSTGr	2.56	0.044
pTFusCr-TOFusCl	2.52	0.050

Note. See Table 2 for a key to region abbreviations. NHA = Neurologically Healthy Adults; PWA = Persons with Aphasia.
