# Supplementary figures and images for: Hypoconnectivity of Resting-State Networks in Persons with Aphasia Compared with Healthy Age-Matched Adults
Source: Front Hum Neurosci. 2017 Feb 28;11:91. doi: 10.3389/fnhum.2017.00091 (PMC5329062; doi:10.3389/fnhum.2017.00091)

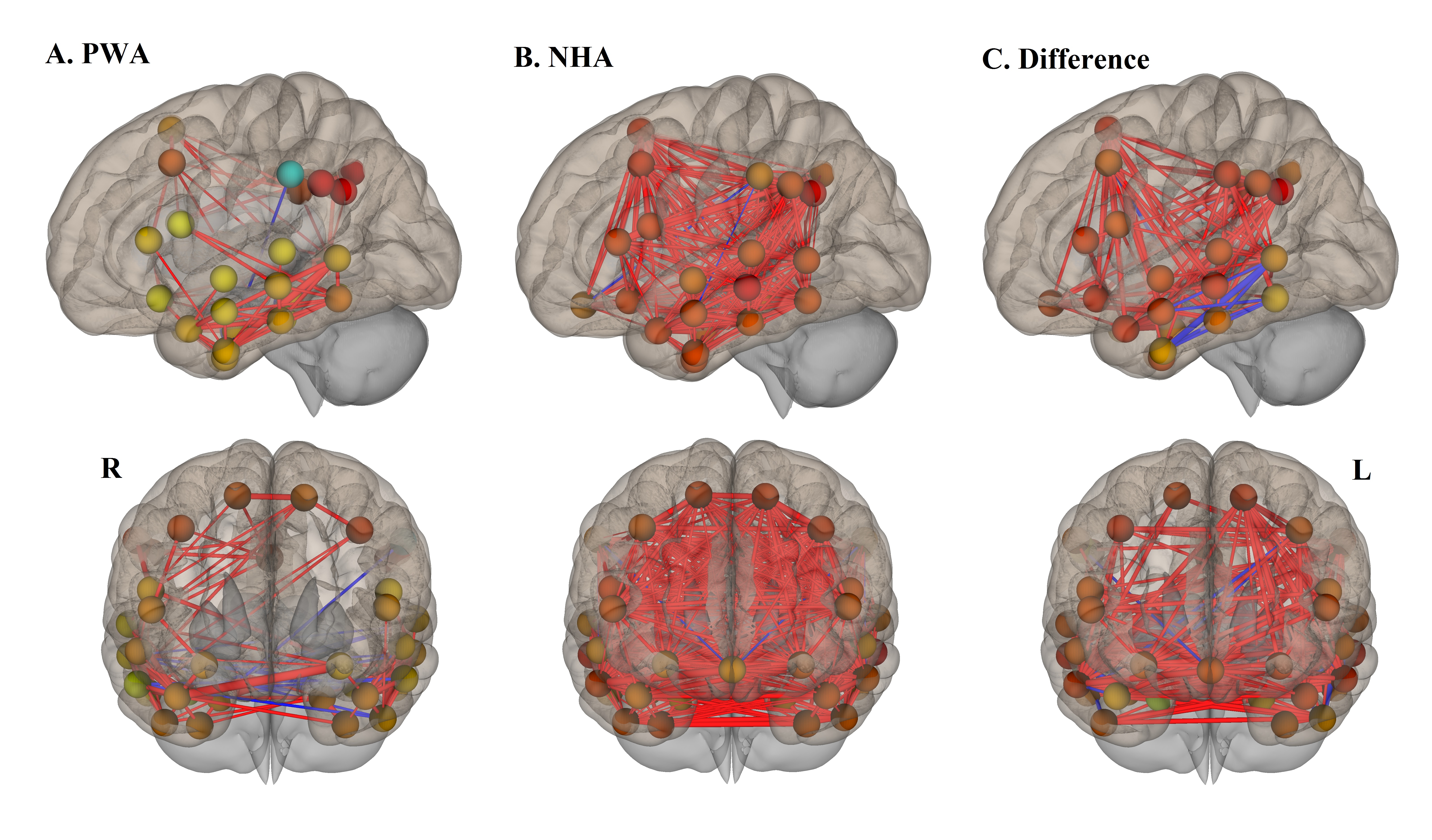

Supplement: Supplementary Figure 1 — Sagittal and coronal views of semantic network. (A) Persons with aphasia (PWA); (B) Neurologically healthy adults (NHA); (C) Difference in connectivity between groups. For (A,B), warm colors represent statistically significant positive correlations, cool colors represent statistically significant negative correlations (anticorrelations). For (C), warm colors represent statistically greater correlations for NHA, cool colors represent statistically greater correlations for PWA. See Supplementary Tables 1, 2 for exact T-values and FDR p-values. [file Image1.JPEG]
